# Energy and institution size

**DOI:** 10.1371/journal.pone.0171823

**Published:** 2017-02-08

**Authors:** Blair Fix

**Affiliations:** York University, Faculty of Environmental Studies, Toronto, ON, Canada; Utrecht University, NETHERLANDS

## Abstract

Why do institutions grow? Despite nearly a century of scientific effort, there remains little consensus on this topic. This paper offers a new approach that focuses on energy consumption. A systematic relation exists between institution size and energy consumption per capita: as energy consumption increases, institutions become larger. I hypothesize that this relation results from the interplay between technological scale and human biological limitations. I also show how a simple stochastic model can be used to link energy consumption with firm dynamics.

## 1 Introduction

Throughout the last century, there has been a recurrent desire to connect human social evolution to changes in energy consumption [1–4]. The motivation is simple: the laws of thermodynamics dictate that any system that exists far from equilibrium must be supported by a flow of energy [5]. Since human societies are non-equilibrium systems, it follows that energy flows ought play an important part in social evolution. However, it has proved difficult to move from grand pronouncements based on the laws of thermodynamics to a *quantitative* understanding of the relation between energy use and social evolution [6]. This paper offers a contribution to such a quantitative understanding.

This paper is concerned with one particular aspect of social change: the growth in size of the *institutions that control human labor*. While such institutions have taken many forms throughout history, in the modern era, the control of human labor is dominated by two institutions: the *business firm* and *government*. In this paper, institution *size* refers to the amount of human labor (i.e employment) controlled by an organization. Under this definition/metric of institution size, I demonstrate that a pervasive, positive correlation exists between institution size and energy use per capita.

I pursue two avenues for understanding the relation between energy and institution size. The first approach draws on the rich history of stochastic modelling within firm size theory. Stochastic (random) models have been successfully used to link firm *dynamics* to the overall firm size distribution. Yet there is little understanding of what drives variations in firm dynamics. Using data on firm age and firm size to constrain a stochastic model, I demonstrate that firm dynamics are likely related to rates of energy consumption, and I offer a prediction of what this relation should look like.

The second approach is more speculative, and aims to offer a general explanation of why rates of energy consumption are related to institution size. I propose two factors that mediate this relation: *technological scale* and *social hierarchy*. I hypothesize that increases in energy consumption involve a trend towards the use of technologies that are larger and more complex. These increasingly large technologies require the coordination of greater numbers of people. Given the limitations of the human brain [7], I argue that large-scale social coordination is most easily achieved through social *hierarchy* [8] and that firms and government are specific manifestations of this hierarchy.

This paper is organized as follows. After a brief review of the strengths and weaknesses of various theories of institutional size (Sec. 1.1), Section 2 discusses the empirical evidence connecting energy consumption with institution size. Section 3 then uses a stochastic model to further illuminate the relation between energy use and firm dynamics. Finally, Section 4 presents and tests a series of hypotheses linking institution size to technological scale and social hierarchy.

### 1.1 Theories of institutional size

Theories of institution size can be divided into two classes: those that concern themselves with the *causes* of institutional growth (‘why’ theories) and those that do not (‘how’ theories). ‘How’ theories have met with great empirical success, while ‘why’ theories have struggled to offer explanations that are testable.

All ‘how’ theories of institutional size can be traced back to the work of the French economist Robert Gibrat, who discovered that the rate of growth of business firms seemed to be *independent* of their size [9]. While later investigation found this ‘law of proportional effect’ to be only approximately true—growth rate variance tends to decline with size [10–12]—it has led to a rich history of stochastic firm growth models [13, 14]. The basic principle is that firm growth is treated probabilistically. Each firm is submitted to a series of random shocks that make it grow (or shrink) over time. When applied to large numbers of firms, the result is a firm size *distribution*. The surprising finding is that these purely random models can very accurately predict the functional form of real-world firm size distributions (see S1 Appendix part F).

Despite their success, ‘how’ theories are not particularly satisfying because they do not explain *why* institutions grow. Unfortunately, theories that *do* attempt to explain the cause of institution growth often rely on unmeasurable variables, and as a result, are untestable.

The theory of the firm has been dominated by Ronald Coase’s *transaction cost* approach. According to Coase, “… a firm will tend to expand until the costs of organizing an extra transaction within the firm become equal to the costs of carrying out the same transaction by means of an exchange on the open market or the costs of organizing in another firm” [15]. Unfortunately, transaction costs have been notoriously difficult to define (let alone measure), rendering Coasian theory untestable [16, 17]

Other theories propose that management talent is the driver of firm growth. For instance, Robert Lucas assumes that the firm size distribution results from “allocat(ing) productive factors over managers of different ability so as to maximize output” [18]. Yet Lucas concedes that the causal factor in this model—the talent of managers—is “probably unobservable”. Despite this problem, Lucas’s theory remains popular [19, 20].

Still other theories propose that firm growth is the result of a resource-driven competitive advantage [21, 22]. Unfortunately, this approach has struggled to stipulate exactly how a particular resource is transformed into a value-creating competitive advantage. Priem and Butler argue that the ‘resource-based view’ advances a theory of value that is tautological—resources create *value* because they are (among other things) *valuable* [23].

In terms of measurability, theories of government size have faired no better than theories of firm size. One approach is to apply the rational-choice model to the behavior of voters. Government size is treated as a reflection of the preferences of utility maximizing voters [24, 25]. However, without an objective measure of individuals’ internal preferences, this theory is untestable.

Another approach is to assume that government bureaucracies (or government as a whole) are self-serving entities that attempt to maximize their budgets, but are restrained by voters and/or an institutional framework such as the constitution [26, 27]. While maximizing behavior is one of the fundamental postulates of neoclassical economics, the hypothesis that humans maximize external pay-offs has been falsified [28].

The lack of measurable variables has consistently plagued ‘why’ theories of institution size. If a new theory is to be successful, it must demonstrate a connection between institution size and some universally measurable quantity. Energy consumption is just such a quantity.

## 2 Energy and institution size: Empirical evidence

To study the relation between energy and institution size, I compare variations in energy use per capita to variations in the size of firms and government over both space and time. For firms, I investigate how changes in the base, tail and mean of the firm size distribution are related to changes in energy use per capita. I use self-employment data to investigate the base of the firm size distribution (relying on the assumption that self-employer firms are very small). To investigate the tail of the firm size distribution, I look at the employment share of the largest firms. To quantify the relative size of government, I measure the government share of total employment.

**Fig 1:**
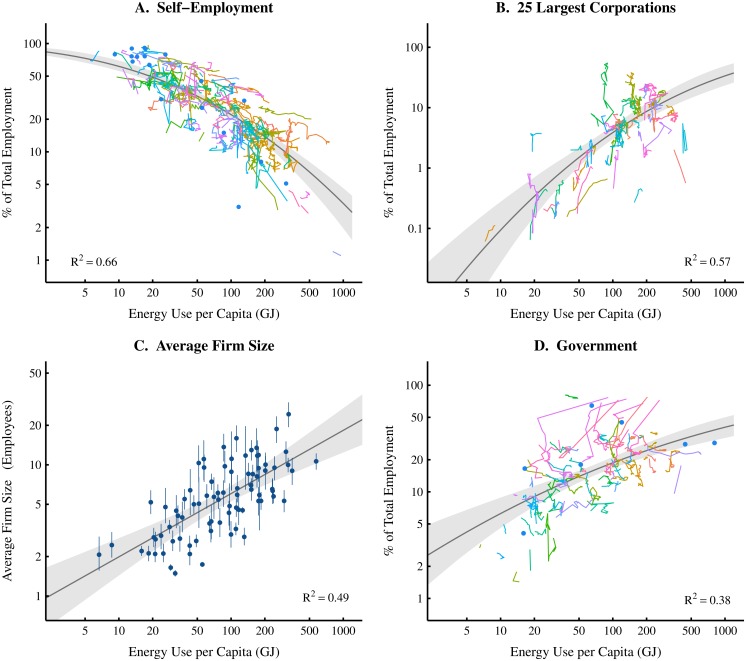
Institution size vs. energy use per capita at the international level. This figure shows how different metrics of institution size vary with energy consumption per capita. Panels A-C analyze variations in firm size by looking at the base, tail, and estimated mean of the firm size distribution. Panel D analyzes variations in government size. In order to show as much evidence as possible, panels A, B and D are a mix of time series and scatter plot. Lines represent the path through time of individual countries while points represent a country with a single observation. Error bars in panel C represent the 95% confidence interval of mean firm size estimates. Variations in self-employment, large-firm, and government employment share vs. energy are modelled with log-normal cumulative distribution functions. Mean firm size vs. energy is modelled with a power law. Grey regions indicate the 99% confidence region of each model. For sources and methodology, see S1 Appendix (part A).

**Fig 2:**
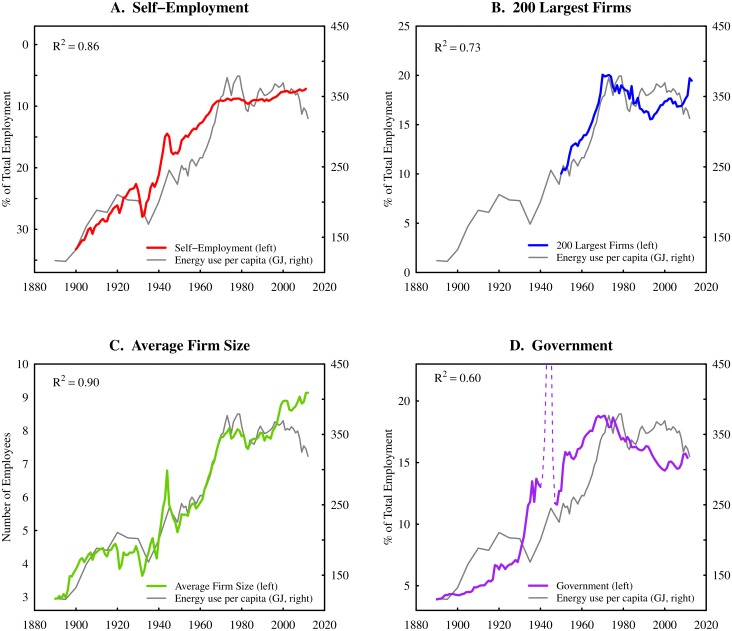
Institution size vs. energy use per capita in the United States. This figure shows the trends for various measures of institution size in the United States over the last century. Trends mirror those found at the global level. As energy consumption per capita increases, self-employment rates decline (panel A, note reverse scale), the large firm employment share increases (panel B), mean firm size increases (panel C), and the government employment share increases (panel D). Note that government regressions exclude World War II (dotted line). For sources and methodology, see S1 Appendix (part A).

**Fig 3:**
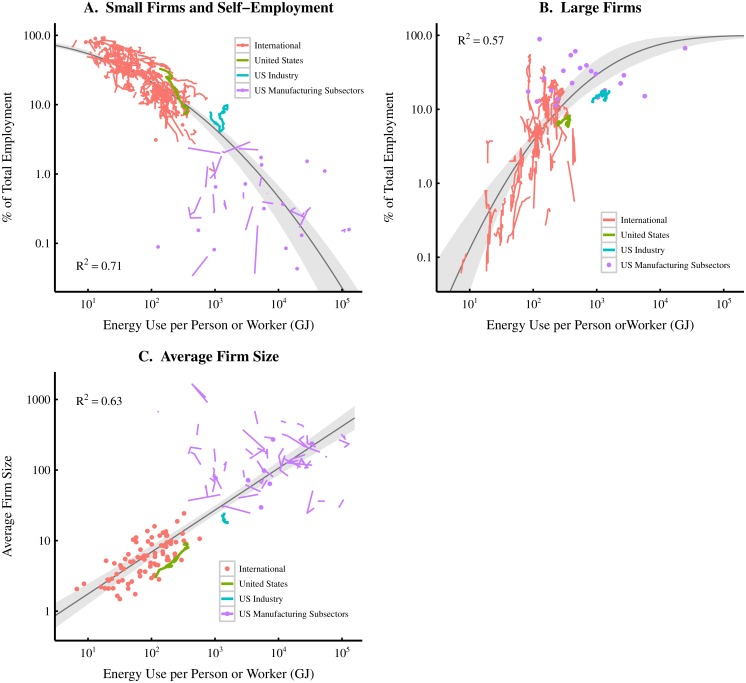
Synthesizing evidence—firm size vs. energy use per person/worker. This figure combines data from 3 different units of analysis (nations, sectors, and sub-sectors) to offer a comprehensive picture of the relation between firm size and energy use per capita (or per worker). ‘US Industry’ consists of construction and manufacturing sectors, while ‘US Manufacturing Subsectors’ are the smallest subdivisions of the manufacturing sector. At the national level, energy use is measured per *person*, while at the sectoral level, it is measured per *worker*. In panel A, self-employment data (for nations and US Industry) is merged with the data for the employment share of firms with 0–4 employees in US manufacturing subsectors. In panel B, data for the employment share of the largest 25 firms (for nations and US Industry) is merged with data for the employment share of firms with more than 5000 employees in US manufacturing subsectors. Panel C shows mean firm size data at the national and sectoral level. Grey regions indicate the 99% confidence region of each model. For sources and methodology, see S1 Appendix (part A).

Comparison of these institution size metrics with energy use per capita are shown in Figs 1–3. International trends are shown in Fig 1 (each colored line represents the path through time of a specific country), while Fig 2 shows time-series data for United States. In Fig 3, I focus only on firms and merge data from Figs 1 and 2 and add US *sectoral* and *subsectoral* level data. Although this synthesis merges data that are not identically defined (see Fig 3 caption), the result is clear: the inclusion of sectoral data serves to extend (by two orders of magnitude) the trends found at the national level. In the case of small firms and mean firm size, the inclusion of sectoral data also increases the regression strength.

To summarize our findings, the evidence in Figs 1–3 suggests the following ‘stylized’ facts. As energy use per capita increases:

1. The small firm employment share *declines*;
2. The large firm employment share *increases*;
3. The mean firm size *increases*;
4. The government employment share *increases*.

Findings 1–3 suggest that increases in energy consumption are associated with a shift in employment from small to large firms. This indicates that the firm size distribution becomes more *skewed* as energy consumption increases. In S1 Appendix (part C), I demonstrate that this shift (at the national level) can be accurately modelled in terms of the changing exponent of a power law distribution.

Assuming a correlation between energy use and GDP, then the evidence presented here is consistent with previous research that has focused on the relation between firm size and GDP per capita [18, 20, 29–31]. However, my focus here on energy use (rather than GDP) is intentional: it is part of a larger effort to ground economic theory in the laws of thermodynamics [32], and to root empirical analysis in biophysical (rather than monetary) phenomena [33–36].

Following the long-standing division in institution size theory between ‘how’ and ‘why’ theories, I adopt two separate approaches for understanding the relation between institution size and energy consumption. The first approach deals with the ‘how’ question: *how* exactly do changes in firm size occur? To answer this question, I use a stochastic model to illuminate the relation between energy use and firm dynamics. The second approach deals with the more difficult ‘why’ question: *why* is institution size related to energy consumption. To answer this question, I investigate the relation between energy, technological change, and social coordination.

## 3 The ‘how’ question: Energy and firm dynamics

Beginning with the work of Gibrat [9] and later Simon and Bonini [37], stochastic models have been successfully used to explain the functional form of the firm size distribution in terms of firm *dynamics*. The implication of these models is that changes in average firm size occur through changes in firm dynamics. Given the connection between energy consumption and firm size, it follows that firm dynamics ought to vary with changes in energy consumption.

Ideally, we would look at this relation directly by investigating international variations in the firm growth rate distribution and comparing them to variations in energy consumption. Unfortunately, data constraints make such a comparison difficult. Calculating international firm growth rate distributions would require longitudinal data for a large, representative sample of firms in many countries. I am not aware of the existence of any such data at the present time. However, we can use what little data *is* available to make inferences about the relation between energy and firm dynamics.

**Fig 4:**
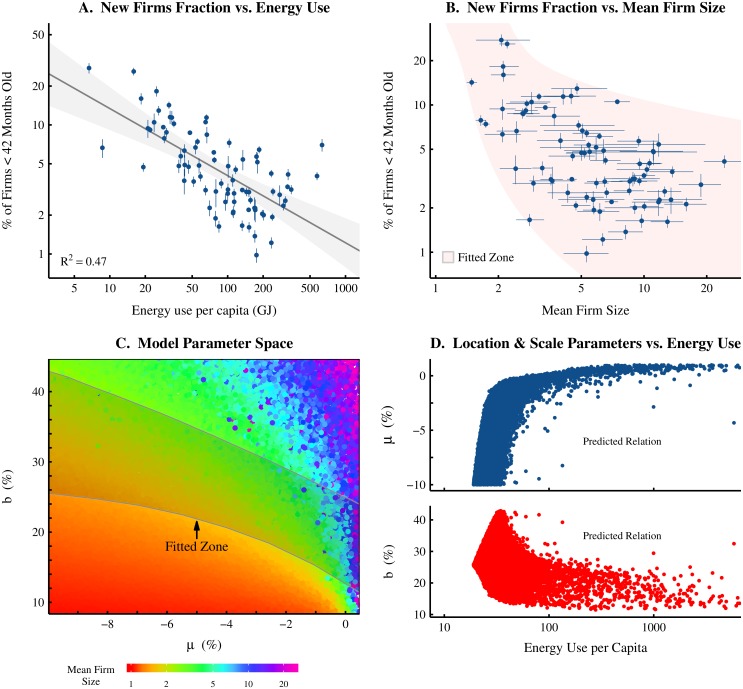
Using firm age data to estimate international firm dynamics. This figure demonstrates how firm age and mean size data can be used to restrict the parameter space of a stochastic model. This allows predictions to be made about the relation between energy use and firm dynamics. Panel A shows the country-level relation between the fraction of firms under 42 months old vs. energy use per capita (the grey region indicates the 99% confidence region of the regression). Panel B shows the country-level relation between the fraction of firms under 42 months old and mean firm size (error bars indicate 95% confidence intervals). The ‘Fitted Zone’ in Panel B shows the age-size relation produced by a stochastic model with a parameter range specifically chosen to capture the empirical data. Panel C shows the model’s parameter space with the resulting mean firm size indicated by color. Using the regressed relation between mean firm size and energy use per capita (Fig 1C), modelled mean firm size is then transformed into an estimate for energy use per capita. The resulting relation between *μ* and *b* vs. energy use per capita (for data in the fitted zone only) is plotted in panel D.

Firm age data provides an indirect window into firm dynamics. If we assume that new firms start at a small size, then we can infer the historic rate of growth of any firm, given its current age and size (i.e. a new, large firm likely grew rapidly, while an old, small firm likely grew slowly). Fig 4A shows how firm age is related to rates of energy consumption per capita. The dataset used here (the GEM database) does not report firm age directly. Instead, it reports whether or not a firm is under 42 months of age. I use this data in Fig 4A to calculate the fraction of firms that are under 42 months of age. This fraction tends to *decline* as energy use per capita increases.

This data clearly hints that a systemic relation exists between energy consumption and firm dynamics. In the following section, I use a stochastic model to make specific predictions about the form of this relation.

### 3.1 A stochastic model

The essence of all stochastic firm models is that growth is treated probabilistically. Each firm begins with some arbitrary initial size *L*_0_. After every discrete time interval, the firm is subjected to a series of random ‘shocks’ (*x*_*i*_) that perturb it from its initial size. In our model, these shocks are drawn randomly from a Laplace distribution. At any point in time, each firm’s size *L*(*t*) is equal to the initial size times the product of all shocks (Eq 1). If the time interval is years, then each shock can be interpreted as the annual growth rate (in fractional form).

$$L\left(t\right)=L_{0}\cdot x_{1}\cdot x_{2}\cdot ...\cdot x_{t}$$ (1)

This basic Gibrat model is unstable unless additional stipulations are added (see S1 Appendix part E). I add a reflective lower bound that disallows firms from shrinking below the size *L* = 1 (this is sometimes called the Keston process [38–40]). As long as firm growth rates have a *downward* drift, the model will produce a stable firm size distribution. Using this model requires the following assumptions:

1. The firm size distribution is a power law.
2. Firm growth rates are independent of size.
3. New firms are all born at size *L* = 1.
4. The firm birth rate is equal to the firm death rate.
5. Firm growth rates come from a *Laplace* distribution.
6. The firm size distribution exists in an equilibrium.

Assumption 1 is necessary because the model produces a power law distribution (see S1 Appendix part F). Recent studies have found that firm size distributions in the United States [41] and other G7 countries [42] are approximately power laws. Less is known about developing countries. In S1 Appendix (part C), I demonstrate that the international data shown in Fig 1 is largely consistent with variations in a power law distribution, as are variations in the US firm size distribution over the last century.

Assumption 2 is a property of most stochastic firm growth models, and dates back to the work of Gibrat [9], who first found evidence that firm growth rates were independent of size. Since then, some studies have found that growth rate volatility tends to decline as firm size increases [10–12]). For the purposes of this model, I neglect this real-world complexity for the following reasons. First, firm growth rate studies use datasets (like Compustat) that are extremely biased towards large firms. Very little is known about the growth rates of small firms. In S1 Appendix (part D), I use the Compustat database (which is very biased towards large firms) to estimate how growth rates might vary with size in a non-biased sample. I find that declines in growth rate volatility are likely important for only a small minority of the largest firms. Furthermore, it is quite possible that the rate at which volatility declines with firm size varies by country and/or through time. However, good data (on which to base a model) is unavailable. Faced with this lack of knowledge, I choose to make the simplifying assumption that firm growth rates do not vary with size.

Assumptions 3 and 4 give meaning to the reflective lower bound. We can interpret this boundary as a firm birth/death zone. Any firm that passes below *L* = 1 is assumed to have ‘died’. The reflection then represents the ‘birth’ of a new firm of size *L* = 1. Since all firms that ‘die’ are immediately ‘reborn’, this mechanism assumes that the firm birth rate equals the firm death rate. This interpretation of the model allows firm age to be defined as the period since the last reflection. In the real world, new firms are obviously not all born at size one; however, evidence suggests that they are much smaller than established firms [43, 44].

Regarding assumption 5, it is well established that the firm growth distribution has a tent-shape that can be modelled with the Laplace distribution [45, 46]. A Laplace (or double exponential distribution) has a sharper peak and fatter tails than a normal distribution. Various theories have been proposed to explain this phenomenon [47, 48]; however the causes of this growth rate distribution are exogenous to the current model.

Assumption 6 justifies testing the model against empirical data. Given some arbitrary initial conditions, the model will always approach a stable firm size distribution that is a function of only the growth rate distribution (provided that the stability conditions are met). Prior to arriving at equilibrium, there is *no relation* between the growth rate distribution and the firm size distribution (since any initial condition is possible). The equilibrium assumption justifies the link between growth rates and the firm size distribution.

### 3.2 Estimating variations in firm dynamics

The goal of this analysis is to estimate how firm dynamics (i.e. growth rate distributions) change with levels of energy consumption per capita. This estimation involves three steps. First, we must use appropriate empirical data to restrict the parameter space of the model. Second, we analyze how this parameter space relates to mean firm size. Finally, we extrapolate, from mean firm size, the relation between model parameters and energy use per capita.

Modelled growth rates are determined by the Laplace probability density function below, where *μ* and *b* are the location and scale parameters, respectively.

$$p\left(x\right)=\frac{1}{2b}e^{-\left|(x-\mu )/b\right|}$$ (2)

The parameter *μ* indicates the most probable growth rate, while *b* corresponds to growth rate volatility (larger *b* indicates greater volatility). Because *μ* and *b* are *free* parameters, we must use appropriate empirical data to restrict their range.

To do this, I use the empirical relation between the proportion of firms under 42 months of age and mean firm size (Fig 4B). A range of model parameters is chosen so that the resulting stochastic model produces the ‘fitted zone’ in Fig 4B. The corresponding parameter space of the model is shown in Fig 4C, with fitted zone parameters indicated by the shaded region. Equilibrium mean firm size for each *μ* and *b* coordinate is indicated by color.

The final step in the analysis is to use the regressed relation between mean firm size and energy use per capita (Fig 1C) to estimate energy consumption levels from modelled mean firm sizes (for data within the fitted zone only). We can then plot the resulting predicted relation between model parameters and energy use per capita (Fig 4D).

Our restricted stochastic model predicts the following: (1) *μ* should *increase* non-linearly with energy consumption; and (2) *b* should *decrease* non-linearly with energy consumption. In general terms, the model predicts that average firm growth rates should increase with energy consumption, while volatility should decline. This result represents a definitive prediction about how firm dynamics should vary with rates of energy consumption. Future empirical work can determine if this prediction is correct.

## 4 The ‘why’ question: Energy, technology and hierarchy

Any attempt to explain why institutions grow must first settle on the appropriate scale: do we attempt to explain why *individual* institutions grow, or do we concern ourselves only with changes in *average* size? The former is almost certainly a futile task, much like offering a general theory to explain why individual species go extinct. The answer is almost certainly, “It is complex”. Species go extinct because of the complicated relation between their physiological characteristics and their environment. Likewise, individual institutions grow/shrink because of the complex relation between their characteristics and their environment (both biophysical and social).

The very success of stochastic firm growth models—in which *randomness* is the explanatory mechanism—suggests that the individual institution is not the appropriate domain for a ‘why’ explanation. Rather, we should be concerned with *groups* of institutions. This decision effectively bars the traditional toolbox of economic theory, which is to construct models based on simple postulates about the behavior of individual entities (consumers, firms, governments, etc.). Instead, we must rely on qualitative reasoning, tested against quantitative empirical evidence.

My explanation of the energy versus institution size relation builds on the ‘social brain’ hypothesis proposed by Dunbar [49]. According to this hypothesis, the size of the human brain inherently limits our ability to maintain social relations. As Turchin and Gavrilets note, social hierarchy offers a way around this limit [8]. Within a hierarchy, an individual must maintain relations with only his direct superior and direct subordinates. This means that a hierarchically organized group can grow in size *without* a corresponding increase in the number of required social relations. I argue that firms and governments are simply the modern embodiment of social hierarchy, and are used as tools of social coordination.

To connect social coordination to energy consumption, I explore the connection between energy use and technological scale. I argue that increases in energy consumption are associated with the use of increasingly large technologies. The construction, operation, and maintenance of these larger technologies, in turn, requires greater social coordination.

I formalize this reasoning in the joint hypotheses below. The order of these hypotheses is meant to show a line of reasoning, not necessarily a direction of causality.

**Hypotheses**
1. Increases in per capita energy consumption are accomplished (in part) through increases in technological *scale*.
2. Increases in technological scale require increases in *social coordination*.
3. Humans have a *limited* capacity to maintain social relations. Hence, egalitarian social coordination has strict limits.
4. *Social hierarchies* allow the scale of social coordination to grow without a corresponding increase in the number social relations.
5. Institutions (firms and governments) are dedicated social hierarchies.

In the following sections, I review the empirical evidence in support of each of these hypotheses.

### 4.1 Energy, technological scale and social coordination

My focus on technology (hypothesis A) is motivated both by theoretical arguments and by the empirical results in Fig 3.

From a theoretical (thermodynamic) perspective, energy ‘consumption’ is best thought of as a *conversion* process. For most organisms, this energy conversion process occurs *within* the body via cellular metabolism. Humans are unique among all other organisms in that we have developed many inorganic ways of harnessing energy *outside* our bodies. This inorganic energy consumption necessarily involves the use of man-made energy converters that transform primary energy into forms useful to humans. We call these man-made energy converters ‘technology’. Since energy use is fundamentally related to technology, it makes sense to explore the ways in which technology relates to institution size.

On the empirical side, the fact that firm size scales with energy consumption both at the national and *sectoral* level (Fig 3) hints that technology mediates this relation. Unlike nation-states, which are defined by geographic boundaries, economic *sectors* are defined by a particular type of *activity*. Similar activities tend to use similar technologies. This is especially true as we move to the smallest manufacturing subsectors. With names like *Sawmills* (NAIC 321113), *Petroleum Refineries* (NAIC 32411), and *Iron Foundries* (NAIC 331511), these subsectors are practically *defined* by the technologies they use. This suggests that differences in energy use between such subsectors are related to differences in the technologies employed.

To illuminate the relation between energy and technology, consider the definitional statement that energy per capita (*E*_*pc*_) is equal to total energy consumption (*E*) divided by population (*P*):

$$E_{pc}=\frac{E}{P}$$ (3)

Let us now define *N* as the total number of energy converters in society. By multiplying by *N*/*N*, we can rearrange Eq 3 to give:

$$E_{pc}=\frac{E}{N}\cdot \frac{N}{P}$$ (4)

Eq 4 indicates that energy use per capita is a function both of technological *scale* (*E*/*N*, average capacity per energy converter) and technological *density* (*N*/*P*, the number energy converters per capita).

In terms of social coordination, there is a fundamental difference between increasing energy consumption through technological density versus technological scale: the former is a *decentralized* process, while the latter requires *centralization*. Increasing energy use per capita through technological density involves independent changes in the behaviour of individuals, meaning it is an atomistic process. However, increasing energy consumption through technological scale requires the centralization of resources and human labor. Thus, it requires increases in social *coordination*.

As an example of a technological *density* process, consider the spread of household appliances (which are a type of end-use energy converter). The invention and widespread adoption of technologies such as the refrigerator, washer, dryer, microwave oven, and dishwasher vastly increased the number of energy converters per capita. At least on the consumer end (not the production end) this process was highly decentralized—individuals *independently* added more electronic devices to their lives.

As an example of a technological *scale* process, consider the changing scale of the industrial technologies shown in Table 1. Relative to their early prototypes, these technologies have undergone increases in scale by factors of one hundred (tanker ships) to factors of over a *million* (electric power plants). These changes in technological scale necessarily involve the increasing coordination of human labor. For instance, the largest oil refinery in the world, located in Jamnagar, India, employs 2500 people on site [50]. Rather than acting autonomously (like the users of consumer electronics), these individuals must coordinate their actions over a wide range of different tasks. This suggests that increases in technological scale require an increase in social coordination.

**Table 1:** Scale increase of various industrial technologies.

Type	Early Prototype	Largest Today	Unit	Scaling Factor
Electric Power Plant	0.0125	2 2500	megawatts	1.80 × 10^6^
Oil Refinery	5.5	1 240 000	barrels per day	2.24 × 10^5^
Aluminium Smelter	5.7	1 060 000	tonnes per year	1.86 × 10^5^
Internal Combustion Engine	0.75	107 390	horsepower	1.43 × 10^5^
Mining Excavator	380	2 324 0000	cubic meters per day	6.12 × 10^4^
Blast Furnace	0.3	5 500	cubic meters	1.83 × 10^4^
Tanker Ship	1809	260 859	gross tonnage	1.44 × 10^2^

This table shows the size of 7 selected industrial technologies at their earliest stage of development (‘Early Prototype’) and at the largest scale existing today. Column 5 shows the scaling factor between the largest and early technologies (largest/early). Technologies are ranked in descending order of scaling factor. For data sources, see S1 Appendix (part A).

**Fig 5:**
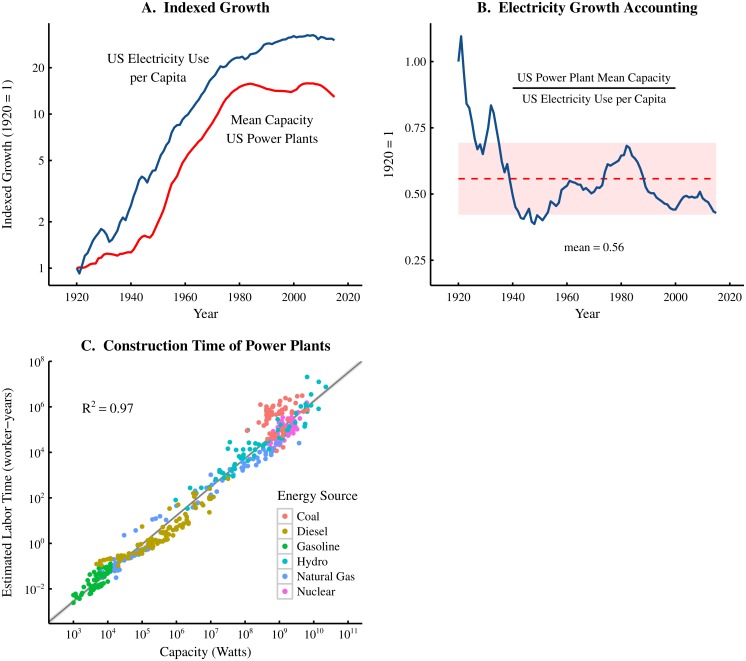
Technological scale and social coordination in electricity generation. Panel A shows the time-series relation between the mean capacity of US power plants and US electricity use per capita. Both series are indexed to 1 in the year 1920 in order to show relative growth. Power plants tend to get larger as electricity use per capita increases increases. Panel B shows the fraction of US per capita electricity use growth (since 1920) that was met by increases in mean plant size. The dashed line indicates the mean over the period 1920–2015, while the shaded region shows the standard deviation. Panel C shows the relation between power plant capacity and the estimated construction labor time. The entire range of electricity generation technology is included in this plot—from the smallest gasoline generators to the largest hydroelectric power plants. Different primary energy sources are indicated by color. Data is modelled with a power law. Grey regions indicate the 99% confidence region of the regression. For sources and methodology, see S1 Appendix (part A).

But to what degree are increases in energy use per capita actually achieved through increases in technological scale? Given the complexity of technological change, this question is difficult to answer at a general level (for all technologies). Instead of a general test of hypothesis A, I present here a case study of electricity production and consumption in the United States (Fig 5A and 5B). The results of this case study indicate that increases in technological scale have played an important role in meeting increases in per capita electricity use over the last century.

Fig 5A shows how the indexed change in US electricity use per capita relates to the indexed change in mean power plant size (as measured by nameplate capacity). Over the last 100 years, the two series tracked together quite closely, with both electricity use and power plant size increasing rapidly between 1920 and 1980 and plateauing thereafter. How important was this change in technological scale for meeting per capita demand? To answer this question, Fig 5B plots the indexed ratio of mean power plant size to electricity use per capita. This ratio indicates the fraction of electricity use per capita growth that was met by increases in power plant capacity. Between 1920 and 2015, increases in power plant capacity accounted for roughly *half* of the total increase in electricity use per capita.

In the US electricity generation sector, increases in technological scale obviously played a major role in meeting increases in per capita electricity consumption. Was this increase in scale accompanied by a corresponding increases in the scale of social coordination (hypothesis B)? Answering this questions requires that we first define what we mean by the ‘scale’ of social coordination, and specify how this relates to a given technology.

I define the ‘scale’ of social coordination as the number of people required to construct, maintain, and operate a specific technology. For measurement purposes, however, I limit my analysis only to *construction* labor time. This decision is driven primarily by data availability (and lack thereof). For the most part, published power plant data focuses almost exclusively on *costs*, and primarily on the cost of *construction*. Fortunately, with a few simplifying assumptions, construction cost data can be used to estimate construction labor time. I use this latter metric to quantify the scale of social coordination associated with a given power plant.

To estimate construction labor time from costs, I first note that by the rules of double-entry accounting, all costs eventually become someone’s *income*. If we assume that all income accrues to labor (i.e. we neglect capitalist income) then we can divide the total cost of a project by an estimate of the average wage to obtain a rough estimate of the total labor time involved. I use GDP per capita as a measure of average income, giving Eq 5 as my method for estimating labor time.

$$\text{Labor}\;\text{Time}\approx \frac{\text{Total}\;\text{Cost}}{\text{GDP}\;\text{per}\;\text{capita}}$$ (5)

Although this method contains some implicit bias/error, I show in S1 Appendix (part G) that it is unlikely that this bias/error affects the integrity of the results (largely due to the vast size range of power plant studied here).

Fig 5C applies this method to estimate the construction labor time of approximately 500 different power plants and generators. The capacity of these plants/generators ranges over 7 orders of magnitude—from the smallest gas-powered generator (1000 watts) to the largest hydroelectric dams (the 22.5 gigawatt Three Gorges Dam). Different energy sources are indicated by color. The results show a strong scaling relation between plant capacity and construction labor time. This indicates that the scale of social coordination necessary to build a power plant is strongly related to the plant’s energy conversion capacity.

To summarize, our case study of the electricity generation sector is consistent with both hypothesis A and B. We find that increases in power plant scale have played an important role in meeting increases in US per capita electricity consumption (hypothesis A). Furthermore, we find that power plant size is strongly related to construction labor time—our measure of the scale of social coordination (hypothesis B).

Admittedly, a case study of a single technology represents limited evidence. However, the vast scaling of the other technologies shown in Table 1 indicates that this line of reasoning has promise. To continue my arguments, I will assume that the findings of this case study can be generalized to many other technologies. The result (we assume) is a that increases in energy consumption require a generalized increase in the scale of human social coordination. The question, then, is how is this coordination accomplished?

### 4.2 Social coordination and human biology

Social coordination can conceivably be achieved in many different ways (customs, markets, institutions, etc.). Thus, an increase in social coordination does not necessarily imply an increase in firm and government size. Why, then, have these institutions increased in size as energy consumption increases? Hypotheses C-E propose a chain of reasoning explaining why institutions are the most effective way of organizing large groups of people. The key to this reasoning is hypothesis C: humans have a *limited* ability to maintain social relations.

The evidence for this hypothesis comes primarily from the work of anthropologist Robin Dunbar, who has uncovered a startling relation between primate brain size and mean group size [7]: primate species with larger brains (as measured by the relative size of the neocortex) tend to live in larger groups. Dunbar has developed this finding into what he calls the *social brain hypothesis*: “primates evolved large brains to manage their unusually complex social system[s]” [49].

The implication of Dunbar’s findings is that the size of the human brain places limitations on the number of social relations that an individual is able to maintain. Dunbar uses his primate data to predict a mean human group size of about 150. While this number should be considered exploratory, Dunbar notes that early egalitarian societies had group sizes around this order of magnitude [51].

A key feature of egalitarian organization is that any member of a group may maintain relations with any other member of the group. Thus, the number of possible social relations increases linearly with group size. Given the hypothesized limitations in the human ability to maintain social relations, it follows that egalitarian social organization is not an effective method for coordinating large numbers of people.

One way of increasing group size beyond Dunbar’s number is to organize groups in a way that *limits* human interaction. Turchin and Gavrilets note that this is a key feature of social hierarchies, which are characterized by a treelike chain of command [8]. Within a hierarchy an individual must maintain social relations only with his direct superior and direct inferiors. Thus, hierarchy allows group size to grow without any corresponding increase in the number of human relations (hypothesis D).

As evidence for this line of reasoning, Turchin and Gavrilets demonstrate that a strong correlation exists between the population of historical agrarian empires and the number of administrative (hierarchical) levels within their respective governments. Similarly, Hamilton et al. find a strong relation between population size and the number of hierarchical levels with various hunter-gatherer societies [52]. This evidence suggests that social hierarchy is a common tool used for increasing the scale of social coordination.

### 4.3 Hierarchy and institution size

Social hierarchies have taken many different forms at different points in human history. For instance, in many pre-state societies, social hierarchy took the form of the chiefdom. In middle-ages Europe, the feudal manor was the principle unit of hierarchy. In the modern era, I argue that business firms and governments are the principle unit of social hierarchy (hypothesis E). To test this hypothesis, I focus only on firms.

The implication of hypothesis E is that increasing firm size constitutes an *investment in social hierarchy*. If this reasoning is correct, then mean firm size should be an indicator of the relative ‘top heaviness’ of a society. Why? Hierarchies tend to become more top heavy as they become larger—the fraction of individuals in the upper echelons tends to grow as the size of the hierarchy increases. Thus, if firms are the modern embodiment of social hierarchy, then mean firm size should be related to the relative size of the upper social echelon.

Since the upper echelons of a hierarchy are almost exclusively involved in managing the activities of other people, it seems sensible to use the *management* profession as a metric for the size of this top cohort. Thus, if hypothesis E is correct, we expect that increases in mean firm size should be associated with an increase in the employment share of managers.

**Fig 6:**
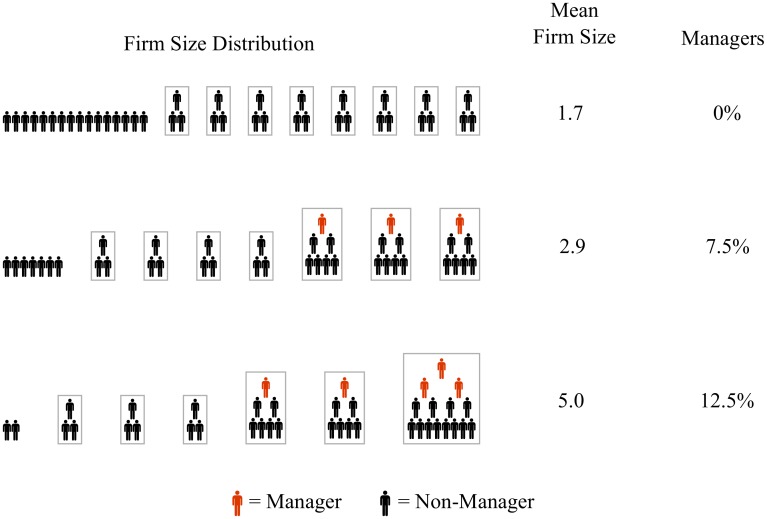
The growth of management as a function of the firm size distribution. This figure graphically demonstrates how the management fraction increases with firm size (assuming firms are ‘ideal hierarchies’). Firms are indicated by boxes (with the exception of single-person firms) with a worker’s hierarchical position shown vertically. The span of control—defined as the size ratio between adjacent hierarchical levels—is constant for all firms. In this picture, the span of control is 2. Managers (red) are assumed to be all individuals in and above the third hierarchical level. To maintain simplicity, this graphic does not use a power law firm size distribution.

To refine this prediction, I develop a hierarchical firm model of society (Fig 6) based on the following assumptions:

1. All firms are ‘ideal’ hierarchies with a single span of control.
2. All individuals in and above the third hierarchical level are considered ‘managers’.
3. The firm size distribution is a power law.

Why assume that management begins at the third hierarchical level? Obviously, individuals within the lowest hierarchical level have no management responsibilities. Those in the second hierarchical level can be thought of as ‘working supervisors’—individuals who have some supervisory responsibilities but who spend a majority of their time engaged in ‘production’ [53]. I assume that individuals in and above the third hierarchical level are devoted mostly to managing the work of others.

This model predicts that the management fraction of employment should grow non-linearly with firm size, eventually approaching an asymptote defined only by the span of control. If the span of control is *s*, then the asymptote occurs at 1/*s*^2^ (see S1 Appendix (part H) for the details of this calculation).

**Fig 7:**
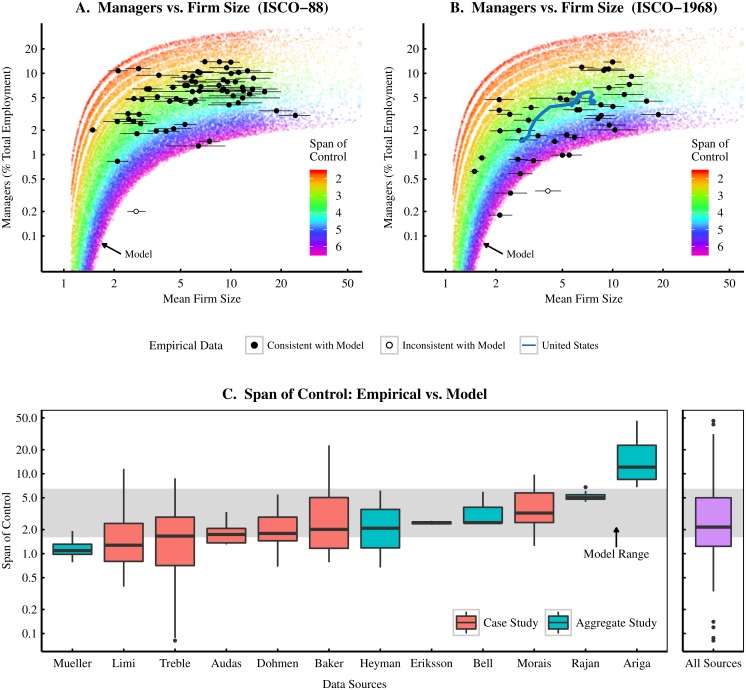
Testing the hierarchical model of the firm using managment share of total employment. Panels A and B plot the country-level relation between the management fraction and mean firm size. Modelled data is also shown in the background, with the span of control indicated by color. Panels A and B use different (incommensurable) classification methodologies for ‘management’. Panel A uses ISCO-88 (which includes legislators, senior officials and managers) while panel B uses ISCO-1968 (which includes administrative and managerial workers). Error bars indicate the 95% confidence intervals for mean firm size. Panel C compares the span of control range from the model to the span distribution found by 12 different empirical studies. Red boxplots indicate case studies, and show the span of control distribution within a *single firm*. Blue boxplots indicate aggregate studies and show the span of control distribution across many different firms. The span of control distribution across all 12 studies is shown on the right. For sources and methodology, see S1 Appendix (part A).

In Fig 7 I test this model at the international level. Fig 7A and 7B plot the country-level relation between the management fraction of employment versus mean firm size (the two plots show different occupation classification regimes). Empirical data is shown in black, while model predictions are shown in the background with the span of control indicated by color. Different mean firm sizes are produced by varying the exponent of the firm size power law distribution (for a technical discussion of this model, see S1 Appendix part H).

The model nicely reproduces the observed relation between mean firm size and the management fraction of employment. However, this fit is achieved by freely manipulating the span of control parameter. Thus, it is important to check that the modelled span of control range is consistent with the span range for *real* firms.

Ideally we would be able compare the span range of the model to the span distribution of a large, global sample of firms. Unfortunately, data constraints make this impossible. Due to the proprietary nature of firm personnel data, only a handful of studies have analyzed firm hierarchies. Fig 7C shows data from 12 such studies that together sample firms from 7 different nations (Denmark, Japan, Netherlands, Portugal, the United Kingdom, the United States, and Sweden). The resulting firm sample gives relatively good coverage of wealthy nations, but unfortunately does not include any firms from developing countries (due to the lack of available studies). For a summary of the data sources, see S1 Appendix (part A).

Boxplots in Fig 7C correspond to the span of control range found by each study. Note that the data is a mixture of case studies of single firms and aggregate studies that analyze the structure of many different firms. While these aggregate studies give better scope than the case studies, many focus only on the upper levels of the hierarchy (where data is more easily obtained). The important finding in Fig 7C is that the model’s fitted span of control range is consistent with the available empirical data.

To summarize these findings, a simple hierarchical firm model of society is able to replicate the observed relation between mean firm size and the management share of employment. The changes in mean firm size are achieved by varying the exponent of a firm size power law distribution, while the management fraction of employment is fitted by ‘tuning’ the span of control range (assumed to be the same both within and between all modelled firms). Importantly, the resulting fitted span range is consistent with the existing empirical data on the internal structure of the firm. The success of this model gives support to hypothesis E, and suggests that increases in mean firm size are characteristic of a generalized increase in social hierarchy.

### 4.4 Causality

I have proposed hypotheses A-E as a chain of reasoning connecting energy consumption to institution size. But which way does causation run? Do increases in energy consumption *cause* institutions to become larger, or is the reverse true? As I discuss below, it seems likely that causation runs in *both* directions.

Although hypotheses A-E are framed in terms of *increases* in energy use (and institution size), I think that a discussion of causation is clearer when framed in terms of *constraints* and *decline*. For instance, I think it *must* be the case that energy constraints place limits on institution size. This is for the simple reason that energy conversion technology is *useless* without an energy input. I have proposed that large institutions provide the social coordination necessary to build and operate large technologies. But without sufficient energy input, these technologies cannot be operated, and the institution’s raison d’être ceases to exist. Imagine how long a large steel firm would stay in business if there was not enough coke to fuel its large blast furnaces. This line of thinking implies that a decline in energy consumption (due to scarcity) can *cause* a decline in institution size.

**Fig 8:**
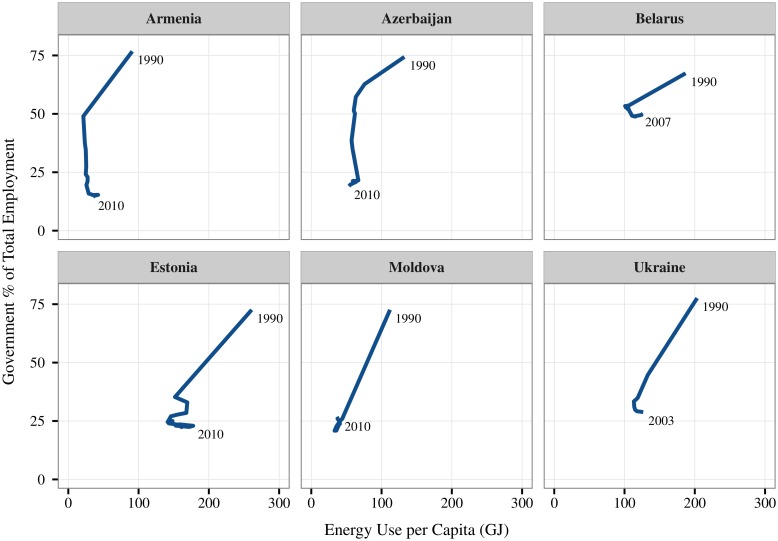
A case study in causality: The collapse of the soviet union. This figure tracks the path through time of six nations that emerged after the collapse of the Soviet Union (in 1990–91). As the collapse unfolded, the fraction of people employed by the government shrank rapidly, as did energy use per capita. Since the USSR collapse was an institutional crisis (not an energy crisis), this suggests that at least in this case, causality runs from institution size to energy consumption.

However, recent history (the collapse of the Soviet Union) suggests that causality can operate in the *reverse* direction. Fig 8 shows energy and government employment share trends in six nation-states that emerged after the dissolution of the USSR. In the aftermath of the Soviet collapse, these six countries experienced *drastic* reductions in both government size and energy use. During this period, there was no global energy shortage, meaning biophysical energy constraints can likely be ruled out as a causal factor. Instead, it seems likely that institutional collapse is the driving factor here.

This case is illustrative because the Soviet economy relied on an unusually high degree of government control of production, placing an enormous amount of power in the hands of a single institution. Not surprisingly, the collapse of this institution led to social chaos and widespread economic decline. I think this shows quite clearly that institutional collapse can cause a decline in energy consumption.

The argument that causation can operate in *both* directions suggests that energy use and institution size exhibit a *feedback* relation (rather than linear causality). One possible avenue for furthering this research is to use systems modelling. Ugo Bardi has shown that a simple adaptation of the Lotka–Volterra equations can be used to model the relation between energy extraction and a technological stock [54]. A plausible line of future research would be to add institution size to this type of model.

It is also important to note that changes in energy use and institution size occur alongside other social changes, the two most obvious being urbanization and changes in sector composition [35]. It seems likely that these phenomena are all interrelated—part of a complex process of social change accompanying changes in energy consumption. In S1 Appendix (part I), I use an adaptation of the hierarchical firm model (used in Fig 7) to explore the institution size constraints that are inherent in the sectoral composition of agrarian societies. The results offer a promising way of broadening our understanding of why energy use is related to institution size.

## 5 Conclusions

All life on earth is united by a common struggle—a “struggle for free energy available for work” [55]. The ability to harness energy places key constraints on the structure of life, from the level of the cell [56], to the organism [57, 58], to the ecosystem [59]. Within this unifying context, it seems plausible that the structure of human society ought to be related to the ability to harness energy.

Based on this line of reasoning, a branch of scholarship has emerged that studies the role of energy in human societies [36, 60–65]. However, to my knowledge, this paper is the first to explicitly connect energy use with institution size. This connection is important because it is not easily explained by existing institution size theories, which focus mostly on the monetary incentives for institution growth.

I have offered a new theory of institution size that is rooted in human biology, and the theorized limitations of our ability to maintain social relations. I have proposed that institutions (firms and governments) are social hierarchies that serve to increase the scale of social coordination beyond that which is possible through egalitarian relations. I have argued that increases in energy consumption require a general increase in the scale of social coordination, and that increases in technological scale are a plausible reason for this connection. There is, of course, no need for increases in technological scale to be the *only* reason why social coordination increases with energy use—it is simply the easiest to study.

An important prediction of this theory is that increases in energy consumption are associated with a general increase in social hierarchy, meaning power is concentrated in the hands of fewer and fewer people. Although this starkly contradicts neoclassical economic theory, it is consistent with the power-based approach to political economy offered by Nitzan and Bichler [17]. If concentrations of power are at the heart of increases in energy consumption, then the theory developed here may be useful for studying a broad range of modern political economic phenomena.

## Supporting information

S1 Appendix Energy and institution size appendices. Contains information on data sources and methods. It also contains extra analysis referenced in the main paper.
(PDF)

S1 File Data and code. This zip file contains raw and final data for all analysis conducted in this paper. It also includes R code used for modelling.
(ZIP)
