# Supplementary material for: Energy and institution size
Source: PLoS One. 2017 Feb 8;12(2):e0171823. doi: 10.1371/journal.pone.0171823 (PMC5298329; doi:10.1371/journal.pone.0171823)
Supplement: S1 Appendix — Contains information on data sources and methods. It also contains extra analysis referenced in the main paper. (PDF) [file pone.0171823.s001.pdf]

Appendices for Energy and Institution Size

Contents

A Sources and Methodology **3**

B Assessing Size Bias within Firm Databases **10**

C The Firm Size Distribution as a Variable Power Law **20**

D Testing Gibrat's Law Using the Compustat Database **24**

E Instability of the Gibrat Model **28**

F Properties of Stochastic Models **31**

G Bias and Error in the GDP Labor Time Method **34**

H A Hierarchical Model of the Firm **39**

I An Agrarian Model of Institution Size **43**

Acronyms

BEA US Bureau of Economic Analysis

BLS US Bureau of Labor Statistics

EIA US Energy Information Agency

HSUS Historical Statistics of the United States

ILO International Labour Organization

GEM Global Entrepreneurship Monitor

WBES World Bank Enterprise Survey

A Sources and Methodology

Electricity Use per Capita

US electricity use is from HSUS table Db228 (1920 - 1948) spliced to EIA table 7.1, *Electricity End Use, Total* (1949-2015). US population is from Maddison [1] (1920-2009) and World Bank series SP.POP.TOTL (2010-2015).

Energy Use per Capita – International

International energy use per capita data is from the World Bank (series EG.USE. PCAP.KG.OE).

Energy Use per Capita – United States

US total energy consumption is from HSUS, Tables Db164-171 (1890-1948) and EIA Table 1.3 (1949-2012). US population is from Maddison [1] (1890-2009) and World Bank series SP.POP.TOTL (2010-2012).

Energy Use per Capita – US Industry

US Industry energy use is from EIA Table 2.1 (Energy Consumption by Sector). Industry employment is from BEA Table 6.8B-D (Persons Engaged in Production by Industry), where 'Industry' is defined to include Mining, Manufacturing and Construction.

Energy Use per Capita – US Manufacturing Subsectors

US manufacturing sub-sector energy use is from EIA Manufacturing Energy Consumption Survey Table 1.1 (First Use of Energy for All Purposes) 2002, 2006, and 2010. Manufacturing subsector employment is from Statistics of U.S. Businesses (US 6 digit NAICS) for 2002, 2006, and 2010.

Firm Age Composition

The fraction of firms under 42 months old (3.5 years) is calculated from the GEM dataset aggregated over the years 2001-2011 (data series *babybuso*). This series gives true/false values for whether or not a given firm is under 42 months old. Uncertainty in this data is estimated using the bootstrap method [2].

Firm Age Model

In order to model firm age accurately, I use a time step interval of 0.5 years (this allows us to calculate firms under 3.5 years so that we can compare to GEM data). However, most empirical data on firm growth rates are reported with a time interval of 1 year. In order to

facilitate comparison with empirical data, I convert model growth rate parameters (μ and σ) into the equivalent parameters for a time step of 1 year. Code for this conversion process is provided in the supplementary material.

Firm size – International

International mean firm size data is estimated using the Global Entrepreneurship Monitor (GEM) database, series *omnowjob*. Data is aggregated over the years 2000-2011. In order to account for the over-representation of large firms, *I remove firms with more than 1000 employees from the database* (see Appendix B for a discussion).

This 'truncation' amounts to removing the top 0.2% of firms in the GEM database. The effects of this truncation on GEM country samples are shown in Figure 1. For 35 out of 89 counties, this has *no effect*, since these country samples do not contain firms larger than 1000 employees. The median percentage of firms removed (by country sample) is 0.01%. For a small number of countries, this truncation removes more than 1% of firms.

Firms with zero employees are assigned a size of 1. This is an attempt to deal with the ambiguity associated with incorporation. The owner of an incorporated sole-proprietorship is usually treated as an employee (by most statistical agencies), but the owner of an unincorporated sole-proprietorship is not. Both types of firms have a single member.

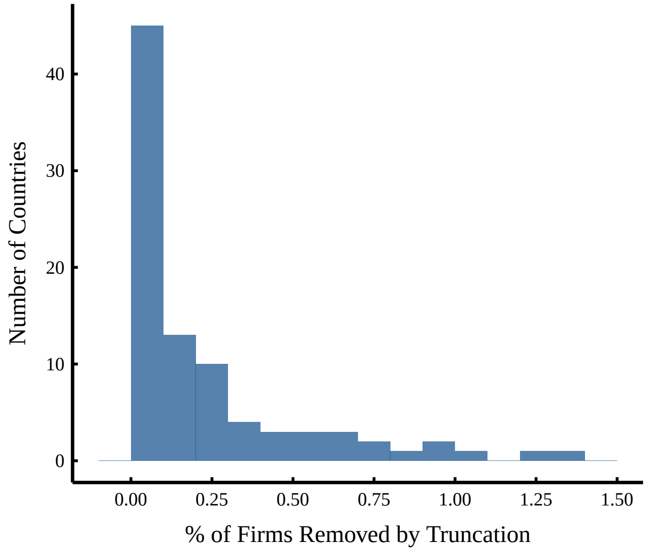

Figure 1: The Effects of Truncating the GEM Database $<$ 1000

This figure plots the country-level distribution of the percentage of firms removed by truncation (firms <1000). The x-axis shows the percentage of firms within each GEM country sample that are removed by truncation. The y-axis shows the number of countries with the given percentage range.

To compare the resulting firm size observations with other time-based series, I use the average year of each country's aggregated data.

Uncertainty in mean firm size is estimated using the bootstrap method [2]. This involves resampling (numerous times, with replacement) the data for each country and calculating the mean of each resample. Confidence intervals are then calculated using the resampled mean distribution.

For comparison between firm size and energy consumption, Yemen and Trinidad are removed as outliers.

Firm size – United States

Average firm size data for 1977-2013 is calculated by dividing the number of persons engaged in production (BEA Table 6.8B-D) by the number of firms. The latter is calculated as the sum of all employer firms in US Census Business Dynamics Statistics plus the number of unincorporated self-employed individuals (BLS series LNU02032192 + LNU02032185).

Average firm size data for 1890-1976 uses firm counts from HSUS Ch408 (which excludes agriculture) and total private, non-farm employment from HSUS Ba471-473 (total employment less farm and government employment). To construct a continuous time-series, the two data sets are spliced together at US Census levels for 1977.

Firm size – US Industry

Mean firm size is calculated using data from Statistics of U.S. Businesses, US 6 digit NAICS and 4 digit SIC between 1992 and 2013. 'Industry' is defined to include Mining, Construction and Manufacturing.

Firm size – US Manufacturing Sub-sectors

Mean firm size is calculated using data from Statistics of U.S. Businesses, US 6 digit NAICS 2002, 2006, and 2010.

Government Employment Share – International

International government employment data is from ILO LABORSTA database (total public sector employment: level of government = Total, sex code = A, sub-classification = 06). Total employment in each country uses World Bank series SL.TLF.TOTL.IN.

Government Employment Share – United States

US government employment data is from HSUS Ba473 (1890-1928), Ba1002 (1929-40), and BEA 6.8A-D persons engaged in production (1940-2011). Total US employment

is from HSUS Ba471 (1900-1928), Ba988 (1929-1940), and BEA tables 6.8A-D (1941-2011).

Large Firm Employment Share – International

The measurement of the large firm employment share is inspired by the work of Nitzan and Bichler [4]. Global data is from Compustat Global Fundamentals (series EMP). Total employment in each country uses World Bank series SL.TLF.TOTL.IN. In some countries, the Compustat data exhibits sharp discontinuities. In order to remove these discontinuities, I have removed the following data: Thailand (1999, 2008, 2010, 2011), Phillipines (2003), Croatia (2011, 2012), and Oman (2010).

Large Firm Employment Share – United States

Data for the largest firms in the United States (ranked by employment) is from Compustat North America, series DATA29 (Figure 2 uses the top 200 firms, while Figure 3 uses the top 25). Total US employment is from BEA tables 6.8A-D (Persons Engaged in Production).

Large Firm Employment Share – US Industry

The employment of the largest 25 firms in US Industry is calculated using the Compustat database, series DATA29. ‘Industry’ is defined to include Mining, Construction, and Manufacturing (all SIC codes between 1000 and 3999). Total Industry employment is from BEA tables 6.8A-D (Persons Engaged in Production).

Large Firm Employment – US Manufacturing Subsectors

Large firm employment share is calculated using data from Statistics of U.S. Businesses, US 6 digit NAICS 2010. ‘Large firms’ are defined here as those with 5000 or more employees. This differs from other data in Figure 3 of the main paper, where the 25 largest firms are used. Figure 2 analyzes the bias in this method. As expected, the number of firms with 5000 or more employees varies significantly by manufacturing subsector. However the median value is 26 firms, meaning that this method should yield similar results to the ‘top 25’ method used elsewhere. There is also no significant correlation between the number of firms with 5000 or more employees, and the sectoral employment share of these firms. Therefore, the variability in the sample size of ‘large firms’ does not cause a directional bias to the employment share of ‘large firms’.

Management Employment Share

Management fraction = management employment / total employment. International management employment is from the ILO LABORSTA database using ISCO-88 (Legislators, senior officials and managers) and ISCO-1968 (Administrative and managerial workers).

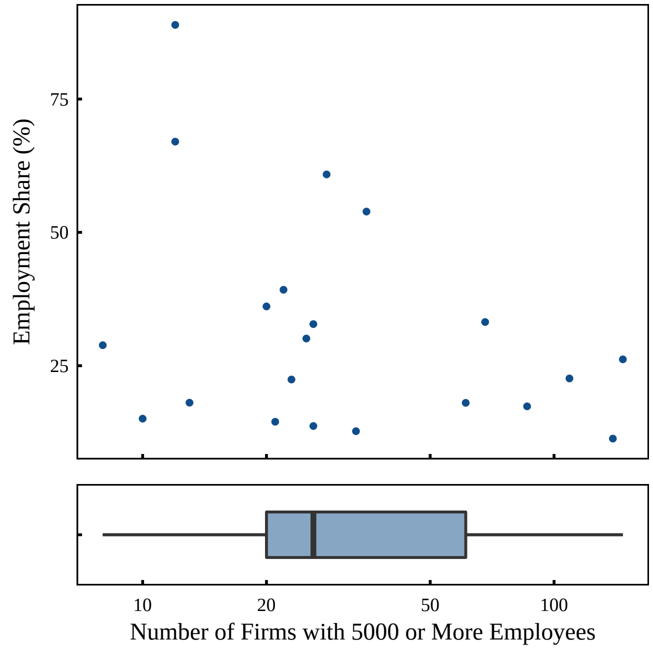

Figure 2: Large Firms in Manufacturing Subsectors — Analyzing Bias Caused by Variations in the Number of Firms

The top panel plots the employment share of 'large firms' versus the number of firms that are defined as 'large' ($\geq$ 5000 employees). Each data point represents a single manufacturing subsector. The bottom panel shows the distribution of the number of 'large firms' per subsector.

Total employment is from World Bank series SL.TLF.TOTL.IN. For ISCO-88, Argentina is removed as an outlier. For ISCO-1968, Syria is removed as an outlier.

US management employment is from BLS Occupational Employment Statistics (various tables, 1999-2014), ILO LABORSTA ISCO-88 (1970-1998) and HSUS Ba1037 (1860-1970). All series are spliced to BLS data. Total US employment is from HSUS Ba1033 (1860-1890), HSUS Ba471 (1900-1928), Ba988 (1929-1940), and BEA tables 6.8A-D (1941-2011). All series are spliced to BEA data.

Power Plants – Construction Labor Time vs. Capacity

Data is compiled by the author from numerous sources. Data and sources are provided in spreadsheet form in S1 File Data and Code.

Power Plants — US Plant Mean Capacity

Plant nameplate capacity data comes from EIA 860 forms from 1990 to 2015. Mean plant capacity counts only power plants that are operational in the given year. Note that form

860 reports *generator* capacity. To calculate *plant* capacity, I aggregate all generators with the same Plant Code.

Self-Employment — International

International self-employment data is from the World Bank, series SL.EMP.SELF.ZS.

Self-Employment — United States

US self-employment data is from HSUS Ba910 (1900-1928), Ba988 (1929-1940) and BEA tables 6.7A–D (1941-2011). Total US employment is from HSUS Ba471 (1900-1928), Ba988 (1929-1940), and BEA tables 6.8A-D (1941-2011).

Self-Employment — US Industry

Industry self-employment data is from BEA tables 6.7A–D. Industry total employment is from BEA tables 6.8A-D (Persons Engaged in Production). Industry is defined to include Mining, Construction, and Manufacturing.

Small Firms — US Manufacturing Subsectors

Small firms are defined as those with 0–4 employees. Data is from Statistics of U.S. Businesses, US 6 digit NAICS 2002, 2006, and 2010.

Span of Control

The span of control is calculated as the employment ratio between adjacent hierarchical levels. Data sources are listed in Table 1.

Technological Scale

Data for technological scale increases (shown in Table 1 of the main paper) is compiled by the author. Sources are available in spreadsheet form in the S1 File Data and Code.

Table 1: Span of Control Data Sources

**Source**	**Ref**	**Years**	**Type**	**N**	**Country**	**Firm Levels**
Ariga	[5]	1981-1989	A	unknown	Japan	All
Audas	[6]	1992	C	1	Britain	All
Baker	[7]	1969-1988	C	1	United States	Management
Bell	[8]	2001-2010	A	552	United Kingdom	Top 3
Dohmen	[9]	1987-1996	C	1	Netherlands	All
Eriksson	[10]	1992-1995	A	210	Denmark	Management
Heyman	[11]	1991,1995	A	560	Sweden	Management
Lima	[12]	1991-1995	C	1	Portugal	All
Morais	[13]	2007-2010	C	1	Undisclosed	All
Mueller	[14]	2004-2013	A	880	United Kingdom	All
Rajan	[15]	1986-1998	A	$\sim$300	United States	Top 2
Treble	[16]	1989-1994	C	1	Britain	All

Notation: Ref = Reference, N = number of firms A = Aggregate Study, C = Case Study

Notes: The 'Firm Levels' column indicates the coverage of the study. 'All' indicates that the study covered all hierarchical levels with the firm(s). 'Management' indicates that only managers were studied. 'Top 2' and 'Top 3' indicate that only the top 2 or 3 hierarchical levels were studied. Raw data from Baker (the BGH dataset) is available for download at http://faculty.chicagobooth.edu/michael.gibbs/.

In many cases, the above papers report results in a table of values, which were then used in this paper. However, some papers report their results only in graphical form. In these cases, I used the *Engauge Digitizer* program to extract data from the graphics.

B Assessing Size Bias within Firm Databases

Like all scientific inquiry, the study of firm size distribution requires reliable data. Unfortunately, accurate firm-size data (with reasonable international coverage) is difficult to find. There are two primary data avenues available: government statistics (the *macro* level) and firm-level databases (the *micro* level). Each avenue has drawbacks.

The problem with relying on macro-level data is that it intrinsically limits the number of countries that can be studied. Apart from wealthy (OECD) nations, reliable macro statistics on firm size distribution are hard to find. This dearth of data often leads researchers to use micro-level databases instead.

The problem with using these micro-level databases to study firm size distribution is that they are rarely (if ever) designed to be *accurate* samples of the wider firm 'population'. As the analysis in this section demonstrates, firm-level databases typically under-represent small firms and over-represent large-firms. Thus, when using a micro database to study the firm size distribution, one must ask: is the database an accurate sample of the firm population? The question that immediately follows is: how do we know if the database is (or is not) biased?

In order to assess database bias, one must inevitably make comparisons to macro-level data. The key is to find macro data that is both relevant and *available* (the second criteria being the more difficult to fulfill). In the following sections I present and apply two methods for assessing firm-size bias within micro datasets.

Methods for Determining Firm-Size Bias within a Database

Method 1: Compare *macro* and *micro*-level average firm-sizes.

Method 2: Compare *micro*-level small-firm employment share to *macro*-level self-employment rates.

Method 1 is straightforward: it involves calculating the average firm-size within a *micro* database and comparing it to the average firm-size calculated from *macro* data. This approach is limited by the availability of macro data. For OECD countries, it is possible to directly compare firm-size averages between micro and macro data. I conduct such an analysis in Table 2 (visualized in Figure 6). Unfortunately, for most non-OECD countries, this approach is not feasible because relevant macro-level data does not exist (hence our need for micro data in the first place).

Method 2 is more indirect (and is dependent on some assumptions); however, its advantage is that self-employment data is readily available for most countries. The basic logic of method 2 is as follows:

1. Self-employed individuals work in *small* firms.
2. We can think of the self-employment rate as an indicator of the share of employment held by the smallest firms.

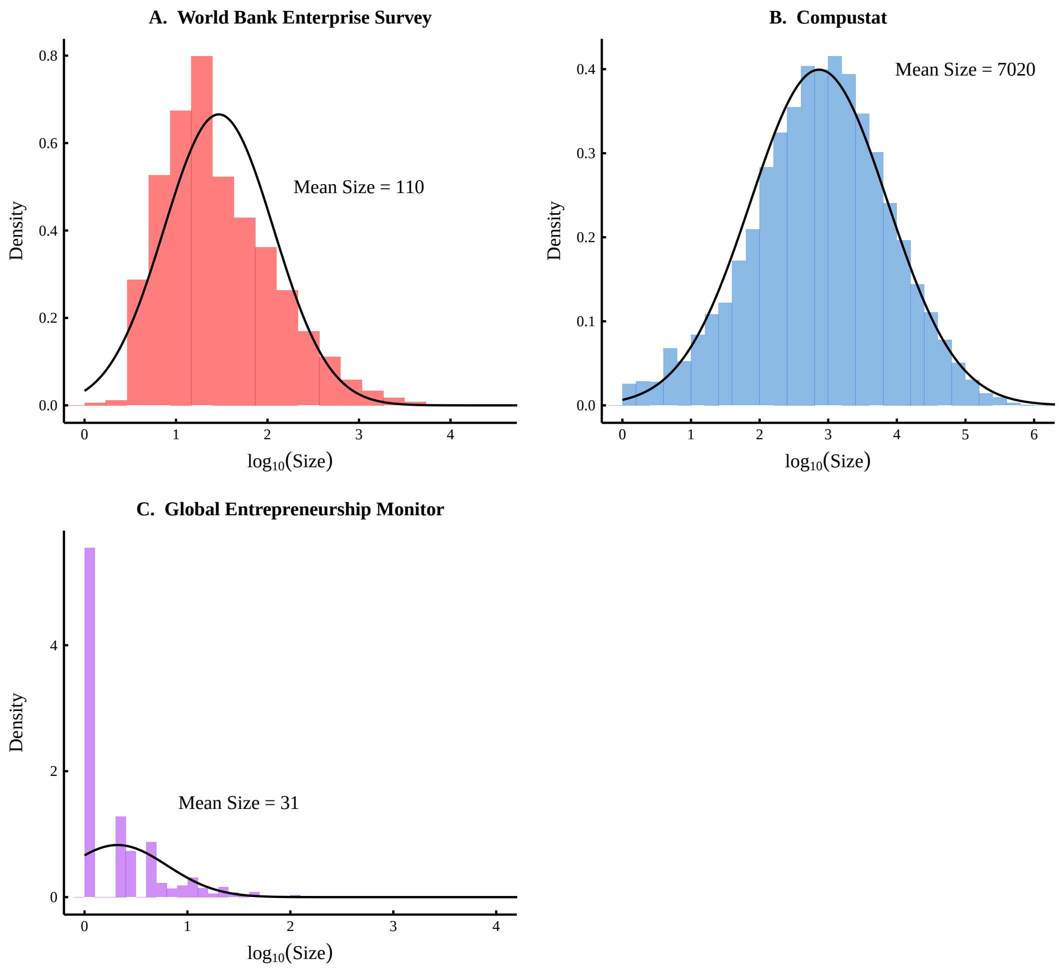

Figure 3: Firm Size Distributions in Selected Micro Databases

Histograms show the firm size distribution within each database (firm size = number of employees). Note that data is log-transformed. Black curves show the best log-normal fit. Panel A shows the firm size distribution of the entire World Bank Enterprise Survery database (for all years). Panel B shows the firm size distribution within the Compustat database (Compustat North America merged with Compustat Global – all available years). Panel C shows the firm size distribution of the Global Entrepreneurship Monitor (GEM) database (from 2000-2011). Note that the log-normal distribution fits both World Bank and Compustat data fairly well, but fits the GEM data very poorly.

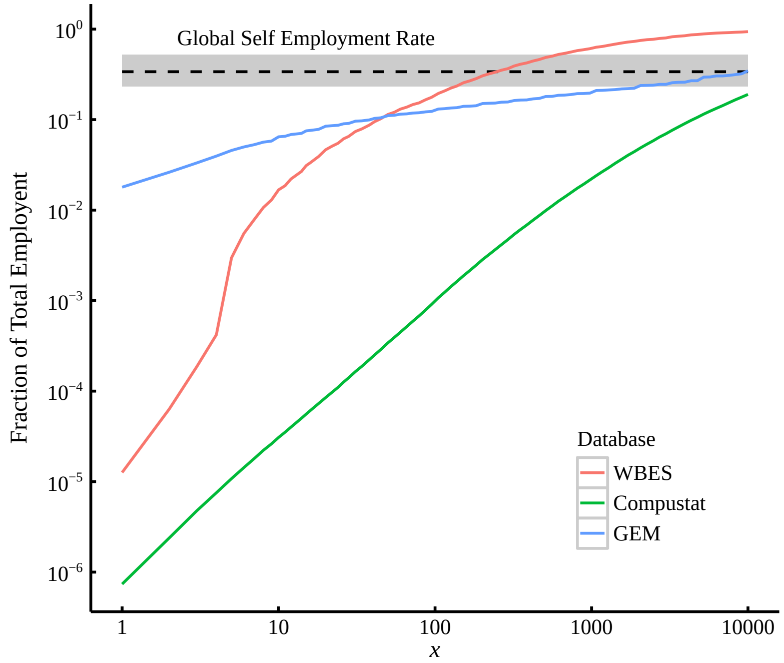

Figure 4: Small Firm Employment Share in Selected Micro Databases

This figure assesses the relative bias within the World Bank Enterprise Survey (WBES), Compustat, and Global Entrepreneurship Monitor (GEM) databases. The share of employment held by firms with x or fewer employees (in each database) is compared to the global self-employment rate between 1990 and 2013 (the dotted line is the median, while the shaded region shows the interquartile range). Sources: Global self-employment data is for self-employed workers who are *non-employers*. This is calculated by subtracting *employer* rates (series SL.EMP.MPYR.ZS) from total self-employment rates (series SL.EMP.SELF.ZS).

---

3. By comparing the self-employment rate to the small-firm employment share within a particular database, we can infer the degree of database bias.

As a starting point, I believe method 2 is more useful, since relevant data is more widely available. In Section B I apply method 2 to three databases: Compustat, the World Bank Enterprise Survey (WBES), and the Global Entrepreneurship Monitor (GEM). Figure 3 shows the firm size distribution within these three databases. The distributions are log-transformed in order to show the log-normal character of two of the three databases (Compustat and WBES).

While all three databases are global in scope, their respective firm size distributions are quite different (note the disparities in mean firm-size). Which database gives the most accurate picture of the underlying population of firms? Analysis reveals that the GEM database is the most consistent with available macro data. Based on these results, in Section B I then conduct a more detailed analysis of the GEM database (see Fig. 6).

Small Firm Employment Share as a Database Bias Test

The basic methodology of this test is to use macro-level self-employment rates as an indicator of the share of employment held by small firms. By comparing this rate to the small-firm employment share within a micro database, we can assess the level of bias.

To begin, we define the small firm employment share as the share of employment held by firms with x or fewer employees (where x is an arbitrary number). We then vary x and see if we can match the resulting small-firm employment share with empirical self-employment rates. Figure 4 conducts such an analysis on the Compustat, GEM, and WBES databases by comparing their respective small firm employment shares to the global self-employment rate.

First, we note that the small firm employment share in all three databases matches global self-employment rates only for a choice of x that is too large to be believably related to 'self-employment'. For WBES, the small firm employment share is similar to the global self-employment rate when x is of order 100. For the GEM and Compustat databases this does not happen until x is of order 10000. This suggests that all three databases have a significant bias towards the under-representation of small firms.

Which database has the least bias? To decide this, we must settle on a believable range for the size of self-employer firms. In the real-world, the boundary x, separating *self-employer* from *employer*, does not exist. However, we can make an educated guess at the likely size range of self-employer firms.

Although a firm size of 1 typically comes to mind when we think of self-employment, the statistical definition of 'self-employment' (as defined by the World Bank) is quite broad. It consists of the following sub-categories:[1]

1. Own-account workers
2. Members of producers' cooperatives
3. Contributing family workers

The inclusion of contributing family workers is important, especially in developing countries where household production is still common. In this context, the size of a self-employer 'firm' will be similar to the size of a family. Since very few families are larger than 10, a believable range for which the small firm employment share should relate to self-employment rates is for $1 \leq x \leq 10$.

Over this range, the GEM small firm employment share is by far the closest to the actual rate of self-employment. While the WBES claims to be a "representative sample of an economy's private sector", this analysis suggests otherwise. The WBES small firm employment share is 2-4 orders of magnitude off the global self-employment rate for $1 \leq x \leq 10$. The Compustat database produces even worse results (off by 4-5 orders of magnitude), but this is expected. Compustat maintains records only for *public* corporations, giving it an inherent bias towards larger firms.

[1] World Bank self-employment data also contains a fourth category called 'Employers'. This category is more aptly called 'owners'. Since firms of all size have owners, I have adjusted the self-employment rate by subtracting the 'Employer' rate.

Note that the WBES and GEM small firm employment shares *cross* at a firm size of roughly 50. Why? The WBES contains very few small firms (size 1-10) and too many *medium* size firms (size 10-50). The GEM database, on the other hand, contains many small firms, but seems to contain too many large firms (size $>$ 1000). This causes the crossing behaviour observed in Figure 4.

This analysis indicates that the GEM database is the most consistent with observed global levels of self-employment. However, it still seems to contain some size bias. The problem, as I discuss in the next section, is that the GEM database contains too many extremely large firms.

Assessing Firm-Size Bias Within the GEM Database

While sufficient to weed out extremely biased databases, the method used in Figure 4 ignores the internal distribution of data within each database. In general, micro databases with global coverage do not contain equal sized samples for each country. Thus, a large, biased sample from one country could potentially skew the entire database, even if other samples are relatively unbiased. To further test database bias, it is important to group data at the national level. In this section I investigate national-level bias within the GEM database.

I begin with a continuation of the self-employment/small-firm method developed above. However, I now group all data at the national level. The GEM database contains firm samples from a total of 89 countries, 72 of which also have data available in the WDI database. For each country, the employment-share of firms with 5 or fewer employees is calculated (from GEM data) and compared to the WDI self-employed rate (non-employers only). This calculation is done for both the full GEM dataset, and a *truncated* version in which all firms with more than 1000 employees are excluded. This truncated version is tested on the hunch that the full GEM database still over-represents large firms (a hunch that is confirmed in Fig. 6).

The results of this analysis are shown in Figure 5. Both the full and truncated GEM databases have a small-firm employment-share distribution that is roughly equivalent to the WDI self-employment rate distribution. Of particular interest is the fact that the small-firm employment share within the truncated GEM database gives a nearly one-to-one prediction of WDI self-employment rates (see Fig 5A).

This analysis suggests that both the full and truncated GEM databases give a reasonably accurate sample of the international firm size distribution. In order to differentiate between the two, it is helpful to compare mean firm-size estimates with macro data. Due to macro data constraints, this must be done with a much smaller sample size than the 72 countries used above. Table 2 shows the 23 countries for which data is available.

Note that macro-level mean-size estimates are predicated on a few assumptions. Government published statistics usually include firm-counts for *employer* firms only (i.e. firms with employees). Non-employer firms are excluded. Thus, unincorporated self-employed individuals are typically not counted as ‘firms’ (incorporated self-employed workers are technically counted as employees of their business, and are thus employer firms). As a result, calculations done using official firm-counts only will give a mean firm-size that is

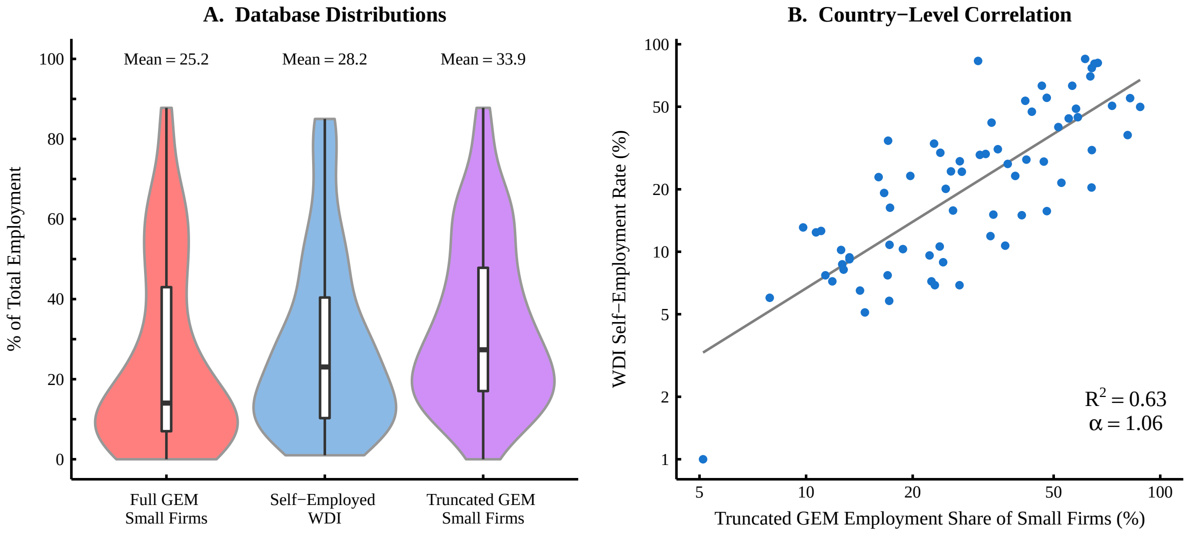

Figure 5: Assessing Small-Firm Bias in the GEM Database

Notes: This figure compares the employment share of small firms ($\leq$ 5 members) in the GEM database to the distribution of self-employment rates (non-employer firms only) within the WDI dataset. Only countries for which data is mutually available are shown (72 countries in total). Unlike Figure 4 all data is aggregated at the national level (countries with small/large sample sizes are all weighted equally). Panel A shows how country-level data is distributed within each database. The 'violin' shows the distribution of data. The internal box plot shows the interquartile range (the 25th to 75th percentile), with the median marked as a horizontal line. Corresponding mean values are shown above. Panel B shows a scatter-plot of country-level data (each point is a country) for the self-employment rate vs. the small-firm employment share in the truncated GEM database. The line shows the best-fit power regression. Note that the regression exponent, α, is nearly 1. Thus, the relation between self-employment rates and small-firm employment share is roughly one-to-one. A similar regression for the non-truncated GEM database (not shown) gives $R^2 = 0.48$ and $\alpha = 0.54$, far from a one-to-one relation. This discrepancy between the full and truncated GEM dataset is the result of the over-representation of large firms within a handful of countries. This skews the small firm employment share downwards (note the low median for the full GEM database in Panel A). Thus, the truncated GEM database is more consistent with self-employment data, meaning we can infer that it has less of a firm-size bias.

Sources: Non-employer rates are calculated by subtracting employer rates (series SL.EMP.MPYR.ZS) from the total self-employment rate (series SL.EMP.SELF.ZS). WDI data is chosen for which the data year most closely matches the GEM year (which is calculated as the country-level mean year of all data entries from 2000-2011).

Table 2: Mean Firm-Size in the GEM Dataset vs. Macro Data

Country	Macro	GEM Trunc	GEM Full
Austria	7.6	11.7	12
Belgium	5.6	6.3	6
Czech Republic	3.5	13.5	30
Denmark	9.2	8.5	26
Finland	6.8	5.3	13
France	7.5	5.3	22
Germany	10.4	11.9	151
Hungary	5.7	6.1	8
Italy	3.5	2.8	17
Netherlands	6.1	10	27
Poland	4.7	2.9	16
Portugal	3.6	8.9	9
Russian Federation	18	9	16
Slovakia	4.2	11.8	17
Slovenia	4.9	13	19
Spain	5.5	4.5	10
Sweden	5.8	5.7	15
Switzerland	10.8	6.5	180
Turkey	3	9.5	18
United Kingdom	7.7	7	26
United States of America	9.1	10	164
India	2.6	5.2	6
Ghana	1.5	2.2	2
Mean	6.4	7.7	35.2

Notes: This table compares mean firm sizes within the GEM database to macro-level data. Data is shown for both the full GEM database, and its truncated version, which removes all observations of firms with more than 1000 employees. The rational for truncation is that large firms are over-represented within the dataset, skewing mean firm-size.

Sources and Methodology: Macro-level mean firm-size is calculated by dividing total employment by the number of firms. The number of firms N_{total} is calculated using Eq. (1), where N_{gov} is government data for the number of firms, S_T is the self-employment rate, S_E the self-employed *employer* rate, U is the fraction of self-employed firms that are unincorporated (hence not counted in official statistics), and L is the size of the labor-force.

$$N_{total} = N_{gov} + (S_T - S_E) \cdot U \cdot L \tag{1}$$

Data for S_T, S_E and L come from World Development Indicators (WDI) series SL.EMP.SELF.ZS, SL.EMP.MPYR.ZS, and SL.TLF.TOTL.IN, respectively. Data for the official number of firms comes from OECD Entrepreneurship at a Glance 2013. Due to lack of data, U is assumed to be 0.7, the level observed in the US [17]. For Ghana, all data comes from Sandefur [18], Table 1 and 2. For India, all data comes from Hasan and Jandoc [19], Table 1 and Table 3 (using the sum of the ASI and NSSO datasets). For US data sources, see Appendix A.

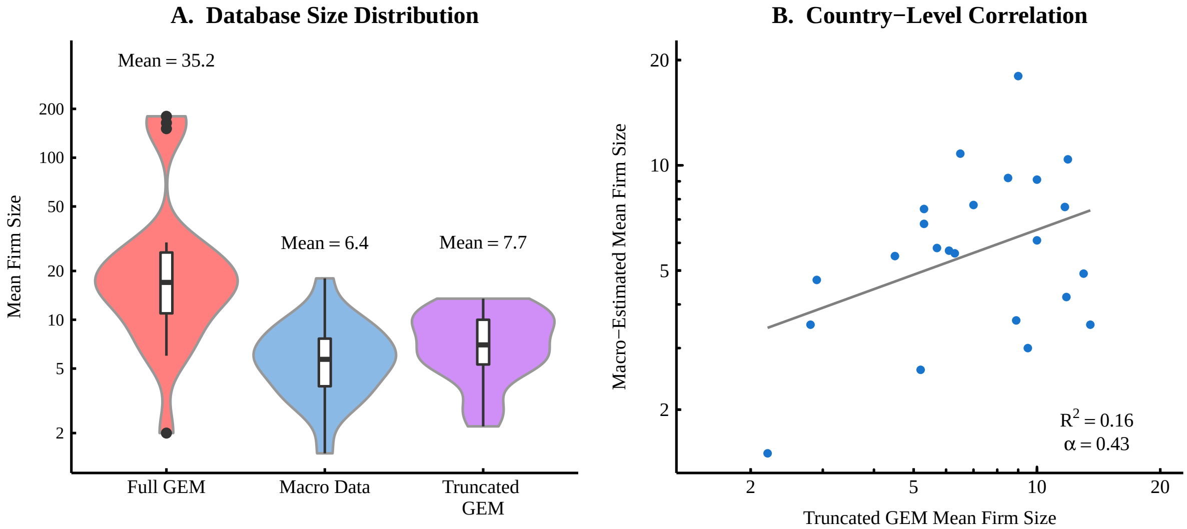

Figure 6: GEM Mean firm size distribution vs. Macro Data

Notes: This figure visualizes the mean firm-size data for the countries shown in Table 2. Panel A shows the mean firm-size distribution within each database. Relative to macro data, the full GEM database clearly over-represents large firms. The mean firm-size in the truncated GEM database is also slightly larger than the macro data, but given the small sample size, the difference is statistically insignificant ($p = 0.20$). Panel B shows the correlation between macro data and the truncated GEM data. A power regression gives an exponent $\alpha = 0.47$, below the desired one-to-one level that would indicate perfect agreement between the micro and macro data. Despite these shortcomings, the truncated GEM database appears to be a fairly accurate sample of the international firm-size distribution.

---

disproportionately large. To account for this bias in macro data, I adjust the official firm-count by adding an estimate for the number of self-employer firms (see the methodology in Table 2).

The results of this investigation are visualized in Figure 6. From this analysis, there is convincing evidence that the full GEM database over-represents large firms. For a few countries (Germany, Switzerland, and the US) this leads to a mean firm-size estimate that is a factor of 10 larger than macro estimates. Truncating the GEM database seems to effectively adjust for this bias.

Why is truncation effective (and is it justified)? The problem of firm-size bias is partially due to the extremely skewed nature of the firm size distribution. The presence of even a *single* extremely large firm can have a large effect on the mean of a sample. For instance, the GEM database contains roughly 170,000 observations. Suppose that the mean firm-size of these observations is 5. If we add a single observation of a Walmart-sized firm (2 million employees), the resulting average more than *triples* (to roughly 17). Of course, firms this large do exist, but the chance of observing one in a sample should be *extremely* small.

The fact that large firms are over-represented in the GEM database demonstrates a sampling bias. Discarding observations of very large firms is one method for dealing with this bias. Other methods are certainly possible, but I do not discuss them here.

Functional Form of the Firm Size Distribution

One of the first tasks for understanding an empirical distribution (of any kind) is to look for theoretical distributions that can be used to model it. Many observers have used the log-normal distribution to model firm size distributions [20, 21, 22, 23, 24, 25]. As shown in Figure 3, the log-normal distribution is a suitable model for the firm size distribution within the Compustat and WBES databases. However, the preceding analysis showed that these databases are rather poor representations of the actual global firm size distribution.

It may be that the use of the log-normal distribution is an artefact of researchers' reliance on biased micro databases [26]. For data that is more representative of the actual firm size distribution (i.e. the GEM dataset), a power law distribution is a much better fit. The characteristic feature of the log-normal distribution is that its logarithm is *normally distributed* (hence the reason for the log transformation in Fig. 3). A power law distribution, however, will not *not* appear normally distributed under a log transformation. Instead, it will decline monotonically as the GEM database does.

Unlike Compustat and WBES, the GEM database is much better fitted with a power law than with a log-normal distribution (see Fig. 7A). For firms under 10,000 employees, the GEM database is consistent with a power law with a scaling exponent $\alpha \approx 1.9$. Note that the tail of the GEM database is 'fatter' than expect for a power law (it is above the 99% confidence interval). This is consistent with our earlier conclusion that the GEM database over-represents large firms. Macro data from for the US firm size distribution is also consistent with a power law (Fig. 7B).

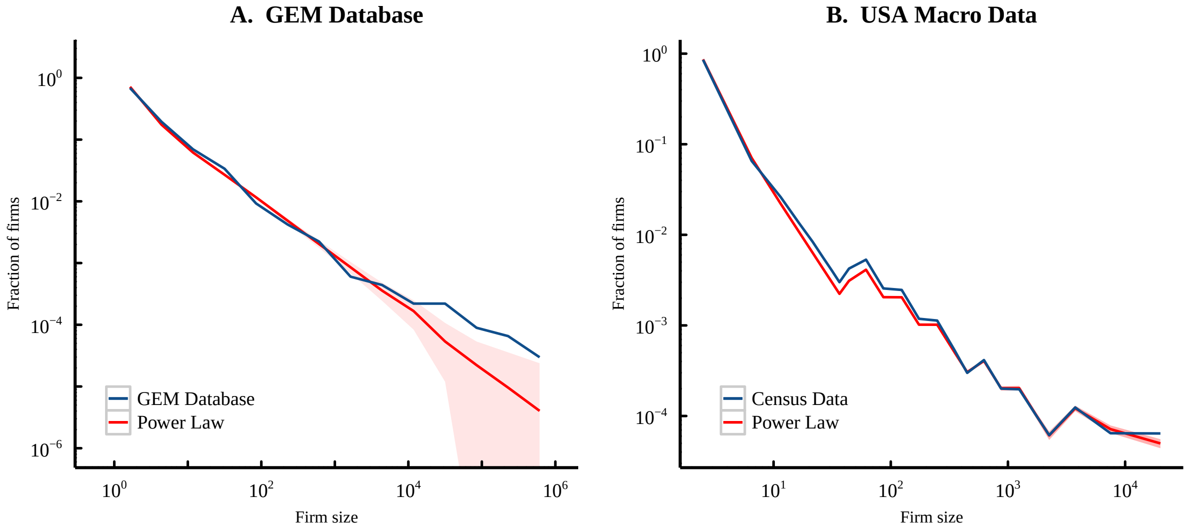

Figure 7: GEM and US Census Data are Consistent with Power Laws

Notes: Panel A shows the firm size distribution of the Global Entrepreneurship Monitor database (all years). For firms with less than 10,000 employees, the database is consistent with a discrete power-law distribution with exponent $\alpha \approx 1.9$. Panel B shows the US firm size distribution, which is consistent with a discrete power-law distribution with exponent $\alpha \approx 2$. Shaded regions show the 99% confidence interval for a simulated power law distribution with a sample size similar to each dataset.

Sources and Methodology: US data for employer firms is from the US Census Bureau, Statistics of U.S. Businesses (using data for 2013). This data is augmented with Bureau of Labor Statistics data for unincorporated self-employed workers (series LNU02032185 and LNU02032192). The histogram preserves Census firm-size bins, with self-employed data added to the first bin. The last point on the histogram consists of all firms with more than 10,000 employees. Both power-law distributions are simulated using the R poweRlaw package, and plotted with the same histogram bins used to plot empirical data. The GEM simulation uses 170,000 observations while the US simulation use 10 million observations.

Note: many readers will expect power law distributions to appear linear when plotted on a log–log scale. Departures from linearity shown in Panel B are artefacts of US census bin sizes (which do not always grow proportionately).

C The Firm Size Distribution as a Variable Power Law

Recent studies have found that firm size distributions in the United States [26] and other G7 countries [27] can be modelled accurately with a power law. Less is known about other countries. In this section, I test if country-level firm size distributions in the GEM database are consistent with a power law. I find that a power law distribution is favored over other heavy-tail distributions in the vast majority of countries. I also find that international variations in 3 summary statistics (mean, self-employment, and large firm employment share) are mostly consistent with a power law distribution.

Power Laws in the GEM Database

The firm size distribution in the *entire* GEM database is roughly consistent with a power law, although the end of the tail is slightly too heavy (Fig. 7A). In this section, I analyse the GEM firm size distribution at the *country* level to assess how well the data fit a power law distribution. I use the truncated GEM database, which contains only firms with fewer than 1000 employees. The rational is that the full GEM database slightly over-represents large firms (see Appendix B).

Historically, power law distributions have been fitted by using an ordinary least-squares (OLS) regression on the logarithm of the histogram. However, this approach is inaccurate, and it violates the assumptions that justify the use of OLS [28]. A more appropriate approach for fitting distributions is to use the *maximum likelihood method*. The likelihood function $\mathcal{L}$ assesses the probability that a set of data x came from a probability density function with the parameter(s) θ.

$$\mathcal{L}(\theta|x) = P(x|\theta) \tag{2}$$

The best fit parameter(s) θ_{mle} maximizes the likelihood function. Like any fitting method, the maximum likelihood indicates only the best fit parameters of the *specified* model, not the appropriateness of the model itself. To discriminate between two different models (1 and 2), we compare their respective maximum likelihoods in ratio form (Λ). The larger likelihood indicates the better fitting model.

$$\Lambda_{1,2} = \frac{\mathcal{L}_1(\theta_{mle}|x)}{\mathcal{L}_2(\theta_{mle}|x)} \tag{3}$$

It is often more convenient to use the log-likelihood ratio, $\log \Lambda$. The sign of $\log \Lambda$ indicates the preferred model (positive indicates that model 1 is better, negative indicates that model 2 is better). The magnitude of $\log \Lambda$ indicates the strength of this preference.

I use this method to assess if country-level firm size distributions in the GEM database are best modelled with a power law. I compare the likelihood of a power law distribution to the likelihood of four other heavy-tail distributions: gamma, log-logistic, log-normal, and Weibull. The resulting range of log-likelihood ratios (one for each country in the GEM database) is shown in Figure 8. A power law distribution is favored over other distributions in the vast majority of countries (97%).

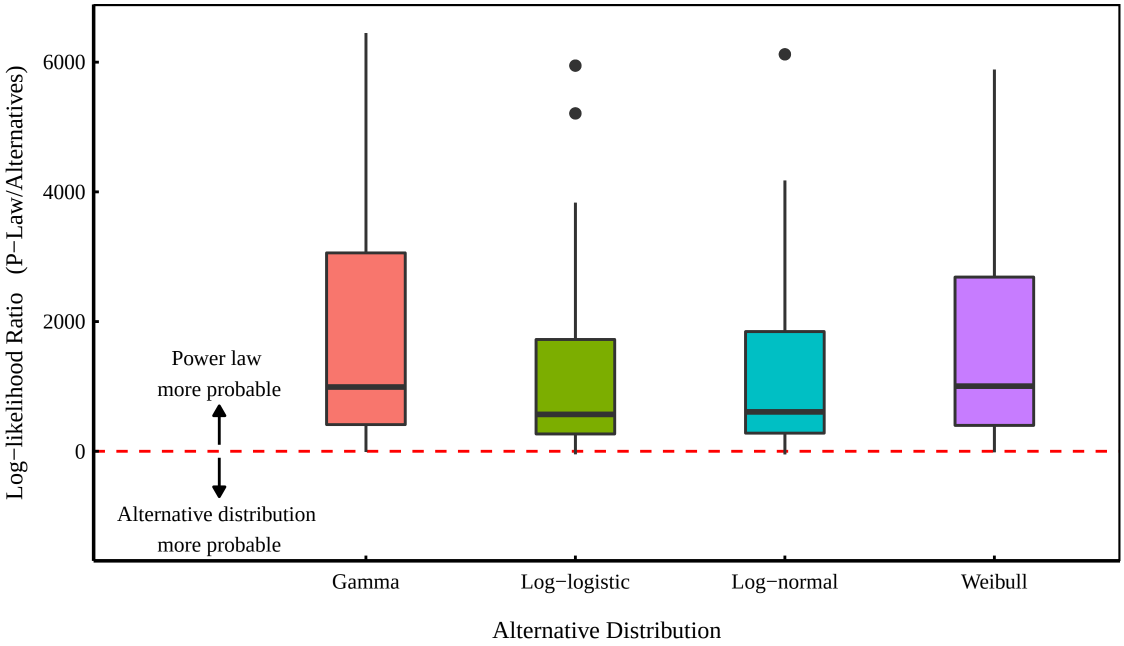

Figure 8: Comparing the Power Law to Alternatives in the GEM Database

Using country-level firm size distributions from the GEM database, this figure assesses the goodness of fit of a power law relative to four other heavy-tail distributions. The firm size distribution in each country in the GEM database is fitted with a power law, gamma, log-logistic, log-normal, and Weibull distribution. For each country, the log-likelihood ratio is computed between the power law and the four alternative distributions. The box plots display the resulting range of ratios. A positive ratio indicates that the power law is more probable, while a negative ratio indicates that the alternative distribution is more probable. In order to better display the majority of data, several large outliers favoring a power law are not shown. For all but 3 countries, a power law distribution is the best fit.

Notes: This figure shows the mean log-likelihood ratios for 100 re-samples (with replacement) of each country. Maximum likelihoods are calculated using the R packages 'poweRlaw' (for a power law) and 'fitdistrplus' (for alternative distributions). Although empirical data is discrete, all models used here are continuous.

International Summary Statistics

Firm size summary statistics can be used as another way to test if the firm size distribution is consistent with a power law. This has the advantage of broadening the evidence to include more data sources (I combine GEM, World Bank, and Compustat data). My method is to pair two statistics and test if the resulting empirical relation can be reproduced by simulated samples from a power law distribution. I look at two pairings: (1) the self-employment rate vs. mean firm size; (2) the large firm employment share vs. mean firm size.

Self-Employment vs. Mean Firm Size

The rational for looking at the self-employment rate is that it indicates the relative share of employment held by small firms. Figure 9A shows the empirical relation between self-employment rates and mean firm size (black dots). The simulated relation is shown in the background, where the power law exponent α is indicated by color. Creating this simulation requires making assumptions about the size of self-employer firms. I assume that *all* firms below the size boundary L_s are considered self-employer firms. The simulated self-employment rate then consists of the fraction of employment held by firms with employment less than or equal to L_s.

To account for international variation in the size of self-employer firms, I let the boundary point vary randomly over the range $1 \leq L_s \leq 10$. In Figure 9A, $L_s = 1$ corresponds to the bottom of the coloured region, and $L_s = 10$ to the top. Why choose the upper bound to be so large? My reasoning is based on the definition of 'self-employment', which consists of 3 sub-categories: own-account workers, cooperatives, and family workers.[2] Especially in developing countries, where household production is still common, a self-employer 'firm' is synonymous with a *family*. A size of 10 seems a reasonable upper limit on the size of family. Given this assumption, a majority of countries (75%), as well as the entire time series for the United States, have a self-employment vs. mean firm size relation that is consistent with a power law.

Large Firm Employment Share vs. Mean Firm Size

To test if variations in the large firm employment share are consistent with a power law distribution, I use the same method as above: I plot the employment share of the 100 largest firms against mean firm size (Fig 9C). I then compare this relation to the one predicted by simulated power law data. To allow for the effects of differing country size, simulation sample sizes vary over the range of national firm populations (which are estimated by dividing the labor force by the mean firm size).

A slight majority of countries (56%), as well as the entire time-series for the United States, have a large firm employment share vs. mean firm size relation that is consistent with a power law distribution. Note that all data points that are *not* consistent with a power

[2] Most statistical databases add a fourth category of 'employers' (i.e. capitalists). Because this category is not related to small firms, I remove it from analysis.

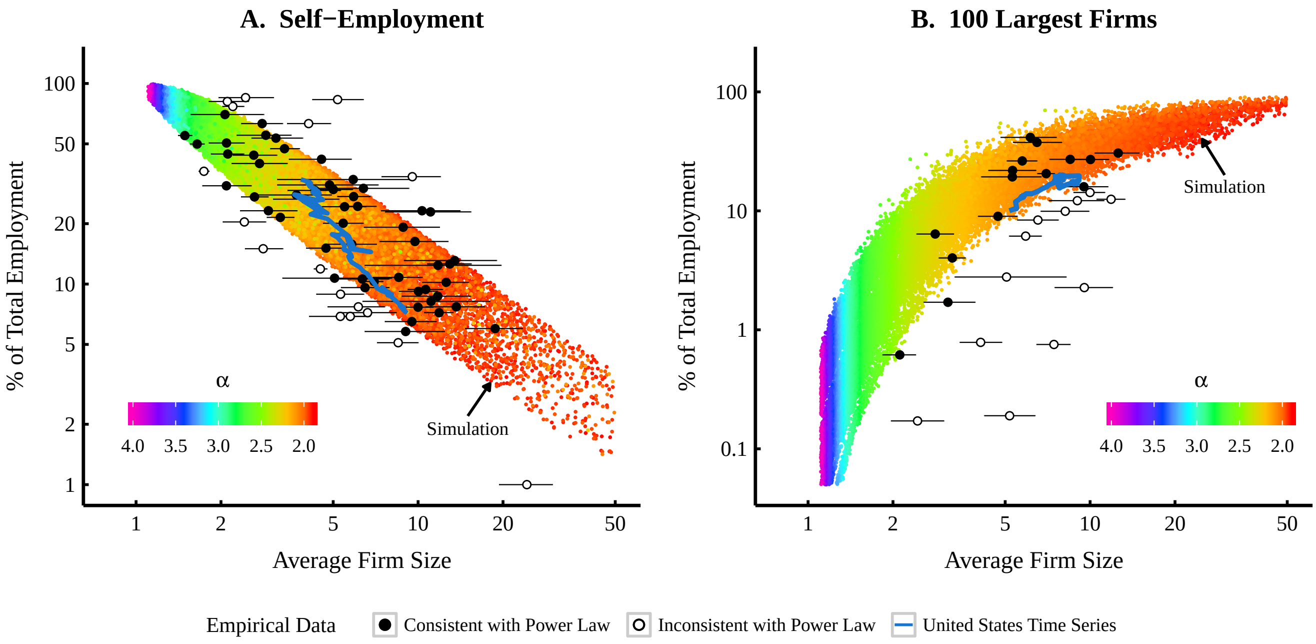

Figure 9: International Summary Statistics, Empirical vs. Power Law

This figure compares pairings of summary statistics for empirical and simulated data. Empirical data is at the country level. Simulated data is randomly generated from a power law distribution (the exponent α is indicated by color). Panel A shows self-employment rates vs mean firm size while panel B shows large firm employment share vs. mean firm size. Self-employment rates are modelled as the employment share of all firms less than the size L_s, which varies randomly over the range $1 \leq L_s \leq 10$. Uncertainty in mean firm size (95% level confidence intervals) is indicated by horizontal lines. Empirical data is judged to be consistent with a power law when the error bar is within the 99% range of simulated data. For data sources, see Appendix A.

law lie *below* the simulation zone (rather than above). This could indicate that these countries have firm size distributions with a tail that is thinner than a power law, but it could also indicate a problem with the data. I have assumed that the 100 largest firms in the Compustat database are actually the largest firms in each nation. There is no guarantee that this assumption is true: the Compustat database may not give complete coverage of the largest firms, especially if a country has many large *private* companies. Further research is needed to determine if these findings indicate a departure from a power law distribution, or if they are artefacts of incomplete data.

D Testing Gibrat's Law Using the Compustat Database

Gibrat's 'law' states that firm growth rates are *independent* of firm size. To what extent is this supported by empirical evidence? I investigate here using the Compustat US database. My results are consistent with previous analysis of the Compustat database: growth rates are approximately Laplace distributed, and volatility declines with firm size [29]. However, I show that this decline is of importance to only a small subset of firms.

Analysis

Rather than directly calculate the mean and variance of Compustat firm growth rates, I fit the growth rate distribution with a truncated Laplace density function (growth rates less than -100% are rounded to -100%). I then investigate how the parameters of this function change with firm size (Fig. 10). The advantage of this approach is that it is not biased by large outliers, and it allows a direct comparison of empirical data to modelled data (where firm growth rates are drawn from a Laplace distribution).

To estimate the Laplace parameters, I fit the histogram of simulated data to the histogram of empirical data (using a Monte Carlo technique that minimizes the absolute value of the error). The results are displayed in Figure 10C-D. The location parameter (μ) shows no significant relation to firm size. However, growth rate volatility (the scale parameter, b) declines monotonically with firm size.

Interestingly, the location parameter is always less than zero, meaning the most probable rate of growth is *negative*. This finding is consistent with the conditions predicted by a stochastic model with a reflective lower bound. Such a model will be stable only when there is a net *negative* drift to firm size (Appendix E). In Appendix F I reproduce the US firm size distribution using a model with a location parameter of -1%, which is consistent with Compustat data.

Extrapolating to the Entire Economy

Because the Compustat database contains data only for publicly traded firms, it is not an accurate sample of the wider US firm population (see Appendix B). However, based on the assumption that the US firm size distribution is a power law, we can estimate how the volatility-percentile relation shown in Figure 10D might look for the economy as a whole. The method for this process is shown in Table 3.

The first step is to generate a US firm sample from a power law distribution that best fits empirical data (I use $\alpha = 2.01$ here), and then compute size percentiles. Next, we select a particular percentile (the green cell) and note the corresponding firm size in the Compustat database (left pink cell). We than find all firms within the power-law sample that have the same size (right pink cells). The scale parameter for the selected Compustat percentile (left purple cell) is then mapped onto these firms, and their corresponding percentiles. The result (right purple cells) is a transformed relation between firm percentile and scale parameter that serves as our economy-wide estimate.

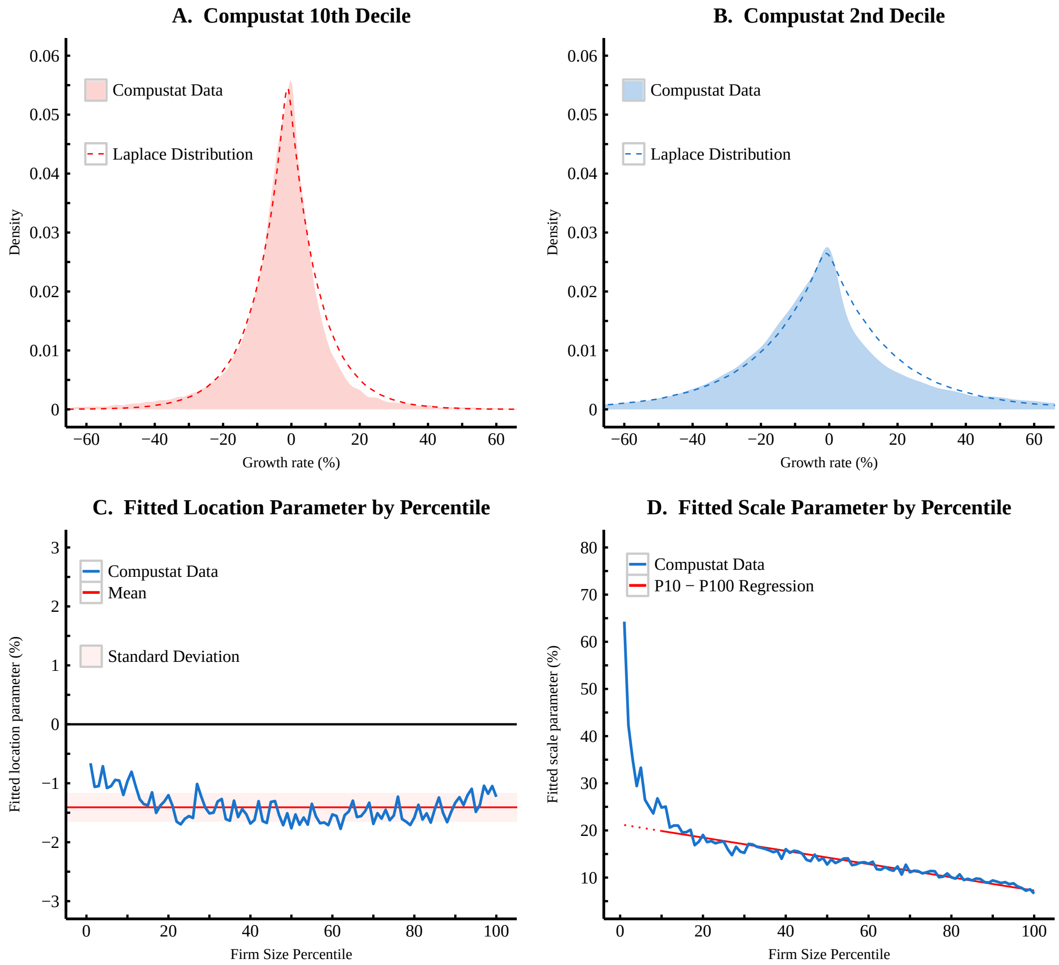

Figure 10: Firm Growth Rate Distribution in the Compustat US Database

This figure analyses firm growth rates (by employment) within the Compustat US database from 1970 to 2013. Panel A shows the growth rate distribution for firms in the 10th (top) decile, while Panel B shows the distribution for firms in the 2nd decile. Dotted lines indicate the best-fit Laplace distribution. Panel C and D show the results of Laplace regressions at the percentile level. Panel C shows the estimated location parameter (μ), while Panel D shows the estimated scale parameter (b). Laplace distributions are fitted using a Monte Carlo method. This analysis indicates that growth rate volatility is a function of firm size, while the growth rate mode is not. Given the firm-size bias of the Compustat database, results for lower percentiles (i.e. P1-P10) should be treated with scepticism.

Table 3: Method for Transforming Compustat Scale Parameter Regressions

Percentile	Compustat Firm Size	Scale	Power Law Firm Size	Transformed Scale
1	1	60	1	60
2	3	50	1	60
3	–	–	1	60
4	–	–	1	60
5	–	–	1	60
6	–	–	2	50

This table demonstrates the method for transforming the Compustat scale-percentile relation to an estimated relation for the whole economy. The first step is to select a percentile (the green cell P1 is selected here). We then match the Compustat firm size of this percentile to the equivalent power law firm size (pink cells). The Compustat scale parameter is then mapped onto all power law percentiles with matching firm sizes, resulting in a transformed scale function (purple cells).

The results of this transformation are shown in Figure 11. Two different estimates are shown. The blue curve shows results using the raw data shown in Figure 10D, while the red dotted curve shows results using a linear regression for P10-100, extrapolated over all percentiles.

Why two different methods? The bias in the Compustat increases as firm size decreases: coverage for large firms is nearly complete, while coverage of small firms (under 10) is extremely limited. Thus, it is quite possible that the large increase in volatility for firm percentiles 1–10 may be an artefact of this bias. By using the linear regression of P10-P100, we remove this potential artefact. We can think of the two curves in Figure 11 as representing a plausible range for the US economy. The stochastic model used to reproduce the US firm size distribution (Fig. 12), has a location parameter of 34%, which is much nearer the lower bound of our Compustat estimates.

This analysis suggest that declines in growth rate volatility are important only to a small minority of firms.

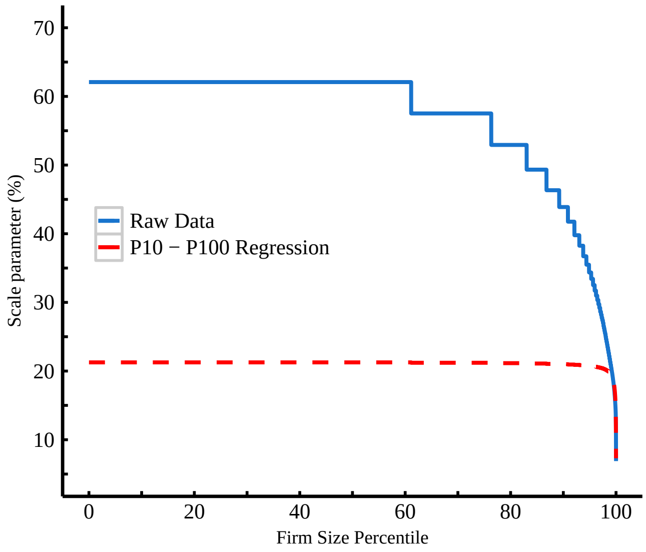

Figure 11: Scale Parameter vs. Percentile, Economy-Wide Estimates

This figure shows a transformation of the Compustat scale-percentile regressions (Fig. 10D) to a form that is consistent with the firm size distribution of the entire US economy. The US distribution is modelled with a power law ($\alpha = 2.01$). The blue curve shows the relation that would result from using the entire range of the Compustat regressions (P1-100). The step-wise pattern is a result of discrete data (steps correspond to a change in firm size by 1). The red dotted curve shows the relation resulting from using a linear regression of Compustat P10-100 (red line in Fig. 10D), extrapolated over P1-10.

E Instability of the Gibrat Model

The Gibrat model assumes that firm growth is a stochastic, multiplicative process. If L_0 is the initial firm size and x_i the annual growth rate, then firm size at time t is given by:

$$L(t) = L_0 \cdot x_1 \cdot x_2 \cdot ... \cdot x_t \quad = \quad L_0 \prod_{i=1}^{t} x_i \tag{4}$$

The instability of this model was first noted by Kalecki [30]. It stems from the model's diffusive nature: the resulting firm size distribution tends to *spread* with time. This tendency can be understood by relating the model to the classic example of diffusion: the one-dimensional random walk.

In a random walk model, a particle is subjected to a series of random additive shocks (y_i) that cause its position to change over time. At any given time, the particle's displacement from the initial position $d(t)$ is simply the sum of all of these shocks:

$$d(t) = y_1 + y_2 + ... + y_t \quad = \quad \sum_{i=1}^{t} y_i \tag{5}$$

In order to intuitively understand how this leads to diffusion, let us suppose that the shocks y_i are drawn from the uniform distribution $\{-1, 1\}$. At any given time, we can ask: what is the maximum possible displacement? In this case, it is exactly equal to t (the number of time intervals that have passed). When we introduce many randomly moving particles, some may attain this maximum displacement (however unlikely it is). Since the maximum grows with time, we can conclude that the displacement distribution must spread with time.[3]

The Gibrat model shares this property, except that the diffusion is *exponential*. To see this, we take the logarithm of Eq. 4, which allows us to express the growth rate product as a *sum*.

$$\begin{aligned} \log(L(t)) &= \log(L_0) + \log(x_1) + \log(x_2) + ... + \log(x_t) \\ &= \log(L_0) + \sum_{i=1}^{t} \log(x_i) \end{aligned} \tag{6}$$

We then exponentiate to get:

$$L(t) = L_0 e^{\sum_{i=1}^{t} \log(x_i)} \tag{7}$$

By setting $\log(x_i) = y_i$, we can see that Eq. 7 is just Eq. 5 in exponential form: our firm growth model is a one-dimensional, *exponential* random walk. The resulting firm size distribution will therefore spread rapidly with time – a fact that is inconsistent with

[3] For a step size drawn from the uniform distribution $\{-1, 1\}$, the standard deviation of the displacement is equal to $\sqrt{t}$. For a good derivation, see Feynman [31] Ch. 6.

available evidence. For instance, we know that the US firm size distribution has changed little since 1970 (see Fig. 2 in main article).

The second problem with this model is that it gives rise to a *log-normal* distribution, contradicting our finding that most firm size distributions are best described by a power law. The proof that this model leads to a log-normal distribution is straightforward. For a sufficiently large number of iterations, the Central Limit Theorem dictates that the sum of independent, random numbers will be *normally* distributed. Thus, for a large number of random walkers, the displacement $d(t)$ will be normally distributed (so long as the distribution of y_i satisfies certain conditions). Because Eq. 7 is the exponential form of Eq. 5, the *logarithm* of $L(t)$ will be normally distributed – the defining feature of the log-normal distribution.

Adding a Reflective Lower Bound

One simple way to reform this model is to add a *reflective lower bound* that stops firms from shrinking below a certain size [32, 33, 34]). This slight change will cause the model to generate a power law, rather than a log-normal distribution. It also leads to model stability (under certain conditions).

Why does the introduction of a reflective boundary lead to a power law distribution? One way of understanding this is to relate back to the additive random walk. If a reflective barrier is added to a one-dimensional random walk, it will no longer tend towards normal distribution; rather, it will tend towards an *exponential* distribution (see [35], p 15 for a proof).

Recall that a multiplicative process can be transformed into an additive process by taking the logarithm. Therefore, for a multiplicative firm model with a lower bound, the logarithm of firm size (L) will be exponentially distributed. Thus, the firm size distribution $p(L)$ is given by Eq. 8, which reduces to a power law (where C is the normalizing constant, and α is the scale parameter).

$$\begin{aligned} p(L) &= Ce^{-\alpha \cdot \log(L)} \\ &= CL^{-\alpha} \end{aligned} \tag{8}$$

For a firm size distribution, the obvious choice for a minimum lower bound is $L = 1$ (a sole-proprietor with no employees). In the proceeding model, I implement this reflection through the following conditional statement, which is evaluated at every time interval:

$$\text{if } \ L(t) < 1, \ \text{ then } \ L(t) = 1 \tag{9}$$

Introducing a reflective lower bound also solves the instability problem, but only when growth rates have a negative 'drift'. Why? Intuitively, we can state that a model will be stable if it is not possible for a firm to shrink or grow *forever*. Introducing a lower bound automatically stops firms from shrinking forever, but it does nothing to stop the possibility of unending growth.

However, if firm growth rates have a net *downward* drift, all firms will tend towards a size of 0, given enough time. This downward drift occurs when the *geometric* mean of the growth rate distribution is less than 1. We can draw an analogy with gas particles moving in a gravitational field on earth. The particles move randomly, but there must be a small net downward drift due to the force of gravity. The result is a stable distribution of particles. If we remove gravity, the particles are free to diffuse forever. Similarly, if we remove the downward bias to firm growth rates, the distribution becomes unstable.

F Properties of Stochastic Models

Despite their simplicity, stochastic models of firm growth are able to replicate many important properties of the real world. I review three such properties here. Stochastic models can be used to:

1. Generate a firm size distribution that is consistent with empirical data;
2. Reproduce the relation between firm size and firm age;
3. Simulate new firm survival rates over time.

Modelling the US Firm Size Distribution

The model used here assumes scale-free growth with a reflective lower bound at a firm size of one. Growth rates are drawn from a Laplace distribution that is truncated by rounding all (fractional form) growth rates less than 0 to 0. In order to maintain a discrete distribution, firms with non-integer size are rounded to the nearest integer (after the application of each growth rate).

This simple model can be used to replicate the US firm size distribution (Fig. 12). In this case, model parameters $\mu = 0.99$ and $b = 0.34$ are used. The model shows the distribution of 1 million firms after 100 time iterations. In order to capture fluctuations around the equilibrium, the model is run 100 times, with the shaded region showing the resulting range of outcomes.

Firm Age vs. Firm Size

Firm age is calculated as the time since a firm's last 'reflection'. The model described above can be used to replicate the size-age relation of firms in the World Bank Enterprise Survey (WBES) database (Fig. 13A). The fitted parameters are $\mu = 0.97, \;\; b = 0.55$. Note that the model diverges from WBES data for firms with fewer than 10 employees. Due to the size bias within the WBES database (see Appendix B), it is not clear if this divergence is significant, or an artefact of database bias.

Firm Survival Rates

The survival rate of new firms tends to decline exponentially over time (Fig. 13B). To replicate this behavior, we give our stochastic model an initial firm size distribution and then track firm survival over time. A firm 'dies' when it is reflected for the first time. At any given time, the firm survival rate is given by the fraction of firms that have never been reflected.

In order to model firm survival rates, we must choose an initial distribution of firms. We can make guesses about this distribution based on BLS *establishment* data. In 1994 — the first year the BLS tracked survival rates — the average size of new establishments was 7.3. In the same year, the average size of *all* US establishments was 16.9 (using data from

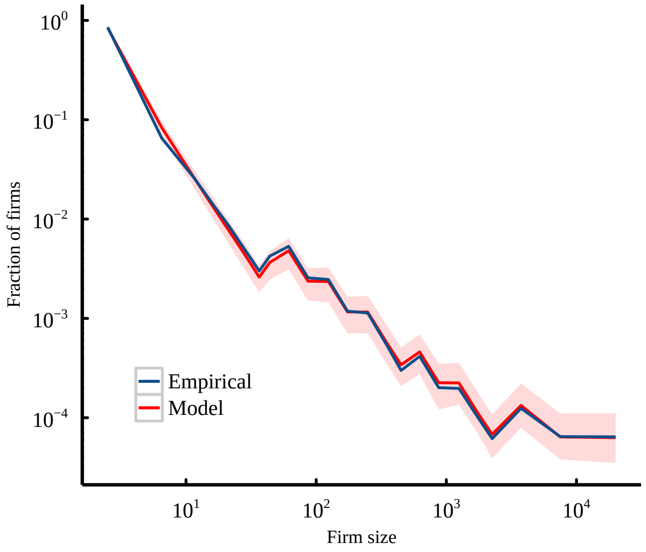

Figure 12: A Stochastic Model of US Firm Size Distribution

The US firm size distribution is shown for the year 2013 (blue line), along with a stochastic model (red) of 1 million firms with growth rates drawn from a truncated Laplace distribution with parameters $\mu = 0.99,\ \ b = 0.34$. The shaded region indicates the 90% confidence region of the model. US Data for employer firms is from the US Census Bureau, Statistics of U.S. Businesses (using data for 2013). This data is augmented with Bureau of Labor Statistics data for unincorporated self-employed workers (series LNU02032185 and LNU02032192). The histogram preserves Census firm-size bins, with self-employed data added to the first bin. The last point on the histogram consists of all firms with more than 10,000 employees. The model histogram uses Census bins to allow direct comparison.

Census Business Dynamics Statistics. It seems reasonable to assume that the average size of new *firms* might also be about half the average for all firms. It also seems reasonable to assume that the distribution of new firms can be modelled with a power law. Using these assumptions, I model the initial firm size distribution with a power law of $\alpha = 2.1$. This gives a mean size of close to 5 (about half the US average).

The empirical data shown in Figure 13 comes from the US Bureau of Labor Statistics (BLS). A caveat is that this data is for *establishment* (not *firm*) survival rates. An establishment refers to a specific business location, while a firm is a legal entity that may contain multiple establishments. For modelling purposes, I ignore this distinction here and assume that establishments are equivalent to firms.

Empirical and modelled survival rates are shown in Figure 13B). The survival rate model parameters ($\mu = 0.99,\ \ b = 0.35$) are nearly identical to the parameters ($\mu = 0.99,\ \ b = 0.34$) used to replicate the US firm size distribution (Fig. 12). These parameters are also consistent with the range estimated from Compustat data (Appendix D).

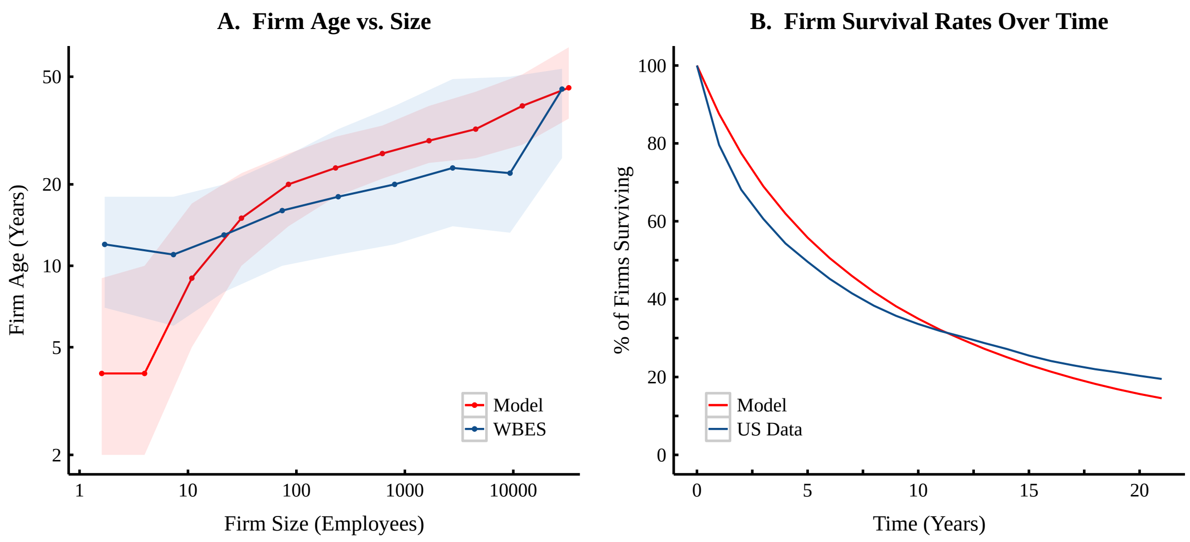

Figure 13: Stochastic Models Can Reproduce Firm Age/Survival Data

Panel A shows the relation between firm size and firm age within the World Bank Enterprise Survey (WBES) database (blue). A stochastic model (red) with growth rates drawn from a truncated Laplace distribution with parameters $\mu = 0.97, \;\; b = 0.55$ produces a similar firm size-age relation. Lines indicate medians and shaded regions indicate the interquartile range. Logarithmic bin locations are indicated with points. Panel B shows the survival rates of new firms over a period of 21 years. Empirical data (blue) is from the BLS Business Employment Dynamics database, Table 7, Survival of private sector establishments by opening year. The model (red) draws growth rates from a truncated Laplace distribution with parameters $\mu = 0.99, \;\; b = 0.35$.

G Bias and Error in the GDP Labor Time Method

My method for estimating power plant construction time is to take total cost and divide by (nominal) GDP per capita in the country and year of construction (henceforth called 'the GDP method'). In this section, I estimate the *bias* in this method. To do so, we need to investigate in detail the assumptions made by this approach.

The total cost of construction (C) of a power plant can be attributed to direct labor costs (L_d), profits and interest (denoted as K, for *capitalist* income), and non-labor costs (N):

$$C = L_d + K_d + N \tag{10}$$

By the rules of double-entry accounting, all non-labor costs will eventual become the *income* of other firms. Thus, after a long digression, we can eventually attribute non-labor costs to either indirect labor costs (L_i) or indirect capitalist costs (K_i):

$$C = L_d + L_i + K_d + K_i \tag{11}$$

Since we are not interested in differentiating between direct and indirect costs, we define L as the sum of direct and indirect labor costs, and K as the sum of direct and indirect capitalist costs:

$$C = L + K \tag{12}$$

Next, we define w as the average wage of all of the workers who are directly and indirectly involved in the construction project. Total labor cost (L) is then the average wage times total labor time (t). Substituting $L = w \cdot t$ into Eq. 12 gives:

$$C = w \cdot t + K \tag{13}$$

Solving for total labor time gives:

$$t = \frac{C - K}{w} \tag{14}$$

Equation 14 gives an accurate estimate of the total labor time involved in construction. Unfortunately, it is difficult (if not impossible) to calculate K (direct and indirect capitalist expenses) and w (the average wage of all direct and indirect workers). In order to get around this lack of data, I make the assumption that capitalist income can be neglected — that labor costs are approximately the same as total costs:

$$L = C - K \approx C \tag{15}$$

Furthermore, I assume that w is approximately the same as nominal GDP per capita (Y_{pc}).

$$w \approx Y_{pc} \tag{16}$$

Under these assumptions, Eq. 14 is approximated by Eq. 17:

$$t \approx \frac{C}{Y_{pc}} \tag{17}$$

By using GDP per capita as a measure of average income, we implicitly assume that all aspects of power plant construction occur within one country. For older plants, this is likely a good assumption. However, in the modern era of globalized production, this assumption is most likely violated to some degree, especially for key components of the plants like the generators and turbines. Unfortunately there is simply no way to disaggregate construction/manufacture costs to their various regions. However, we can correct for this bias to some degree by including power plants from as many nations as possible. The GDP method will then overestimate the labor time for plants constructed in developing countries (where GDP per capita is very low) and underestimate labor time for plants constructed in wealthy countries (where GDP per capita is very high). The hope is that these divergent biases will cancel themselves out.

How accurate is the GDP method? Unfortunately, we cannot compare GDP method estimates to the true labor time value (Eq. 14) because this latter formula contains *unknowable* quantities (K and w). However, we can test Eq. 14 against an alternative estimate for labor time that makes more accurate assumptions.

To proceed, let us first rewrite Eq. 14 as follows by factoring out C in the numerator:

$$t = \frac{C\left(1 - \frac{K}{C}\right)}{w} \tag{18}$$

We then make the assumption that capitalists involved (indirectly and directly) with the project earn profit and interest at approximately the *national average rate*. This means we assume that the capitalist share of total costs (K/C) is approximately the same as the capitalist share of national income (k_s).

$$\frac{K}{Y} \approx k_s \tag{19}$$

We also assume that workers involved (indirectly and directly) with the project earn the *national average wage* (w_n). Given these assumptions, Eq. 18 can be rewritten as:

$$t \approx \frac{C\left(1 - k_s\right)}{w_n} \tag{20}$$

We now have two way of estimating the labor time involved in the construction of a power plant (Eq. 17 and Eq. 20). Our expectation is that Eq. 20 is the more accurate estimate. To quantify the discrepancy between the two estimates, we construct an error ratio, which is the ratio of the two labor time estimates (Eq. 17 / Eq. 20):

$$\text{error ratio} = \frac{w_n}{Y_{pc}\left(1 - k_s\right)} \tag{21}$$

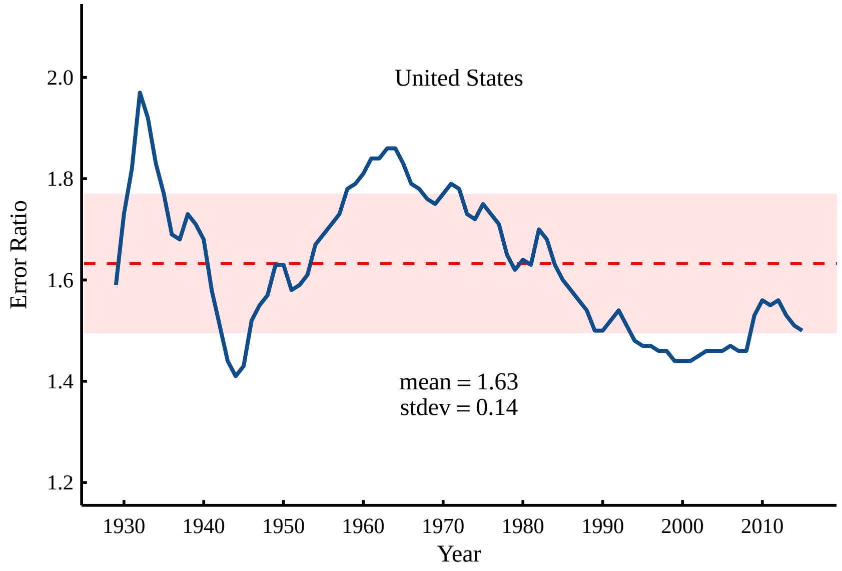

Figure 14: Error estimate of the GDP method for calculating labor time

This figure shows calculations of the error ratio (Eq. 21) using US data. All data is from the BEA. National income data is from Table 1.12, National Income by Type of Income. Capitalist share of national income is equal to profits (with CCA and IVA) and net interest divided by national income. The average wage is calculated by dividing the sum of the compensation of employees and proprietor income by the total persons engaged in production (Table 6.6B-D). US population data is from Maddison [1] and the World Bank series SP.POP.TOTL. Nominal GDP data is from the file *gdplev*.

Figure 14 shows this error ratio calculated using US data from 1929–2015. The results indicate that the GDP method (Eq. 17) *overestimates* labor time by roughly 60%. Why? By neglecting capitalist income, our estimate *inflates* the numerator in Eq. 14. Furthermore, GDP per capita is typically slightly lower than the average annual wage of a full-time worker, so the GDP method *deflates* the denominator in Eq. 14. Of course, this error estimate is itself based on the assumptions contained in Eq. 20. Still, it seems safe to conclude the following:

1. The GDP method likely *overestimates* the true labor time of power plant construction;

2. This overestimate is relatively stable over time.

Since our interest in this study is how labor time *scales* with plant capacity (and not with *absolute* labor time), this constant overestimate is of little concern. It will have no effect on the scaling of construction labor time with power plant size.

What is of more concern, however, are the *changes* in the error ratio that occur over time. How might this affect the estimation of power plant construction time? It is actually quite simple to model the effect of measurement error on a scaling relation. We begin by

assuming that two variables, x and y, exhibit perfect power law scaling *identical* to that found between power plant capacity and construction labor time:

$$y = x^{1.26} \tag{22}$$

To study the effect of measurement error, we introduce a 'noise factor' ϵ (drawn from a lognormal distribution), that perturbs the perfect scaling relation:

$$y = x^{1.26} \cdot \epsilon \tag{23}$$

The effect of larger/smaller error can be modelled by increasing/decreasing the relative dispersion of ϵ. Suppose, for argument's sake, that Figure 14 *severely* underestimates the error associated with the GDP method. In reality, let us assume that the error is *10 times larger*. Since the relative standard deviation of the Figure 14 error ratio is 0.086, we can model the effect of a tenfold increase in error by setting ϵ to have a relative standard deviation of 0.86.

Figure 15 shows how the effects of this error factor (on our power law scaling relation) change as the orders of magnitude spanned by the dependent variable (x) increase. The horizontal axis shows the orders of magnitude spanned by the variable x, while the vertical axis shows the R^2 value of a log-log regression on the relation $y = x^{1.26} \cdot \epsilon$. The important result is that even though the measurement error is quite large, it becomes increasingly *inconsequential* as the data span increases.

Why? The R^2 value indicates the proportion of the variance in the dependent variable (y) that is predictable from the independent variable (x). Since we are conducting a log-log regression, it is helpful to look at the log transformed relation:

$$\log(y) = 1.26 \cdot \log(x) + \log(\epsilon) \tag{24}$$

Now, the variance in $\log(y)$ is affected both by the variance in $\log(x)$ and by the variance in $\log(\epsilon)$. But notice that the variance in both $\log(y)$ and $\log(x)$ will be proportional to the logarithm of the *range* of x. But this is equivalent to the orders of magnitude spanned by x (since orders of magnitude indicate scaling by factors of 10). Thus, the variance in $\log(x)$ and $\log(y)$ scales with the orders of magnitude spanned by x. However the variance in $\log(\epsilon)$ is *constant* — it does not change as the range of x increases. Because the variance in $\log(\epsilon)$ does not scale, its importance decreases as the range of x increases. That is, the fraction of variance in $\log(y)$ that is attributable to $\log(\epsilon)$ is inversely related to the orders of magnitude spanned by x.

So what does this result imply for the accuracy of the GDP method? Clearly, accuracy is a function of the orders or magnitude spanned by plant capacity. In our case study, plant capacity spanned *seven orders of magnitude*. According to Figure 15, even if the GDP method had a *severe* error factor (i.e. only accurate to within a factor of 3), the resulting measurement error would still not have a significant effect on the observed scaling relation. Thus, despite the error that is implicit in the GDP method, it is likely that our results are robust.

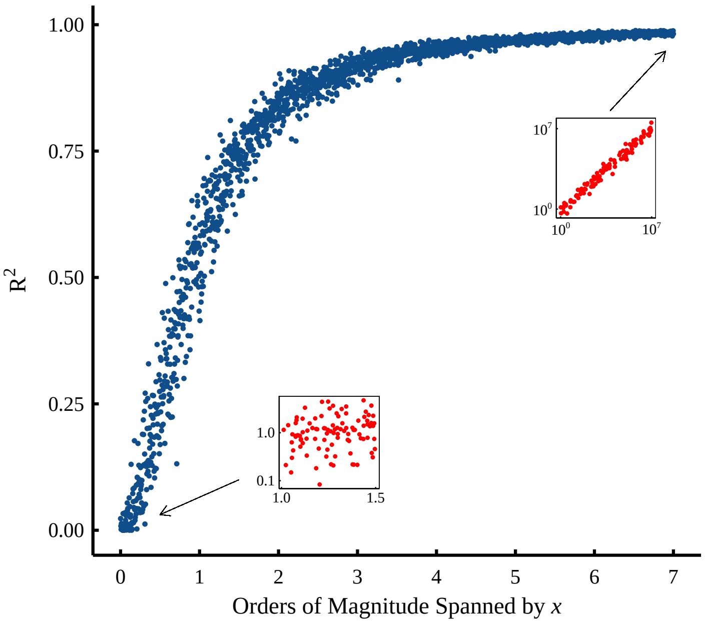

Figure 15: Data span vs. the effect of measurement error on a scaling relation

This figure shows multiple log-log regressions on data defined by the relation $y = x^{1.26} \cdot \epsilon$. Here x is a random variable whose logarithm is uniformly distributed, and ϵ is a noise factor drawn from a log-normal distribution with mean 1 and standard deviation 0.86 (which is 10 times the relative standard deviation of the error ratio in Fig 14). The horizontal axis shows the orders of magnitude spanned by the variable x, while the vertical axis shows the resulting R^2 value of the y vs. x regression. Each dot represents a *single* regression. Inset plots (red) show raw data underlying two different regressions — one with a small data span (bottom left) and one with a large data span (top right). For data that spans less than 2 orders of magnitude, the noise dominates the subsequent regression. However, once the span of x surpasses 4 orders of magnitude, the noise becomes inconsequential to the regression.

Still, given that the GDP method has a bias, why not use the more accurate approach given in Eq. 20? The problem with this formula is that it requires data on the capitalist share of national income as well as data on the average annual income of full time workers. This data is much more difficult to obtain than GDP per capita (especially in developing countries). Thus my use of the GDP method is mostly one of convenience: it makes analysis easier.

H A Hierarchical Model of the Firm

An 'ideal' hierarchy has a constant span of control throughout — meaning the employment ratio between each consecutive hierarchical level is *constant* (Fig. 16). This property allows total employment to be expressed as a geometric series of the span of control s. If the number of individuals in the top hierarchical level is a, and h_t is the total number of hierarchical levels, then total employment L is given by the following series:

$$L = a\left(1 + s + s^2 + ... + s^{h_t - 1}\right) \tag{25}$$

Using the formula for the sum of a geometric series, Eq. 25 can be rewritten as:

$$L = a\frac{1 - s^{h_t}}{1 - s} \tag{26}$$

We make the assumption that individuals in and above the hierarchical level h_m are considered *managers*. The number of managers M in a firm with h_t levels of hierarchy is equivalent to the employment of a firm with $h_t - h_m + 1$ levels of hierarchy:

$$M = a\frac{1 - s^{h_t - h_m + 1}}{1 - s} \tag{27}$$

We can use Eq. 27 and Eq. 26 to express management as a fraction of total employment (M/L):

$$\frac{M}{L} = \frac{1 - s^{h_t - h_m + 1}}{1 - s^h} \tag{28}$$

Asymptotic Behavior of the Management Fraction

The management fraction tends to grow with the number of hierarchical levels, but only to a certain point (Fig. 17). For $h_t > 10$ the management fraction approaches an asymptotic limit that depends only on the span of control s. Finding the asymptotic behavior of M/L requires evaluating the following limit:

$$\lim_{h_t \to \infty} \frac{M}{L} = \lim_{h_t \to \infty} \frac{1 - s^{h_t - h_m + 1}}{1 - s^{h_t}} \tag{29}$$

To evaluate this limit, I use L'Hospital's Rule, which states that $\lim \frac{f(x)}{g(x)} = \lim \frac{f'(x)}{g'(x)}$. We first rewrite Eq. 29 in a differentiable form, with a base e exponent:

$$\lim_{h_t \to \infty} \frac{M}{L} = \lim_{h_t \to \infty} \frac{1 - e^{\log(s) \cdot (h_t - h_m + 1)}}{1 - e^{\log(s) \cdot h_t}} \tag{30}$$

Applying L'Hospital's Rule, we take the derivative (with respect to h_t) of both the numerator and the denominator in Eq. 30, giving:

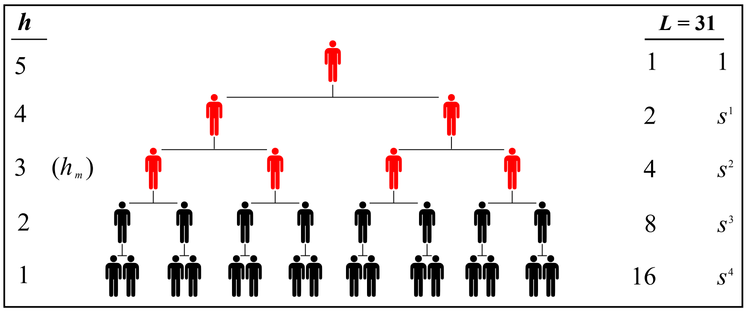

Figure 16: A Perfectly Hierarchical Firm

Within a perfectly hierarchical firm, the number of individuals in adjacent hierarchical levels differs by a factor of the span of control s (in this diagram, $s = 2$). This characteristic allows total employment L to be expressed as a geometric series of s. Managers (red) are defined as all individuals in and above level h_m (which equals 3 here).

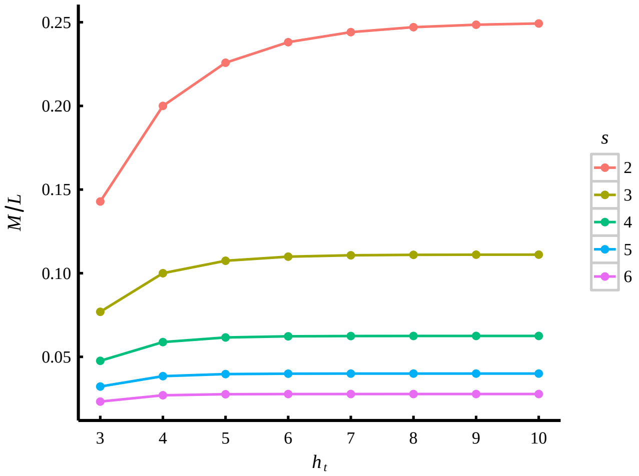

Figure 17: Asymptotic Behavior of the Management Fraction

This figure shows a plot of Eq. 28 for $h_m = 3$ and various s. As the total number of hierarchical levels (h_t) increases, the management fraction (M/L) within a firm grows rapidly, but soon reaches an asymptotic limit. This asymptote is a function of the span of control s, and the choice of h_m (the definition of where management begins).

$$\lim_{h_t \to \infty} \frac{M}{L} = \lim_{h_t \to \infty} \frac{-\log(s) \cdot e^{\log(s)\cdot(h_t - h_m + 1)}}{-\log(s) \cdot e^{\log(s)\cdot h_t}} \tag{31}$$

This simplifies to:

$$\lim_{h_t \to \infty} \frac{M}{L} = e^{\log(s)(-h_m+1)} = s^{-h_m+1} \tag{32}$$

Therefore, the asymptotic behavior of the management fraction depends only on the span of control, and our definition of management.

An Algorithm for Creating Hierarchies

The management model uses a power law simulated firm size distribution. In order to calculate the number of managers, each firm must be organized into hierarchical levels. I have developed the following algorithm to carry out this process.

Having selected a firm, we know its employment L and its span of control s; however, the total number of hierarchical levels h_t is unknown. To calculate h_t, we assume, for the moment, that the size of the top hierarchical level is one. Therefore, h_t must satisfy:

$$L = \frac{1 - s^{h_t}}{1 - s} \tag{33}$$

Solving for h_t gives:

$$h_t = \frac{\log\left[1 + L(s-1)\right]}{\log(s)} \tag{34}$$

Since h_t must be discrete, we round the solution to the nearest integer. My method is then to 'build' the hierarchy from the bottom up. If the bottom hierarchical level contains b workers, then L is defined by the series:

$$L = b\left(1 + \frac{1}{s} + \frac{1}{s^2} + ... + \frac{1}{s^{h_t-1}}\right) \tag{35}$$

Using the formula for the sum of a geometric series, this becomes:

$$L = b\,\frac{1 - 1/s^{h_t}}{1 - 1/s} \tag{36}$$

At the moment, L is known but b is unknown. We therefore solve for b (and round the answer to the nearest integer):

$$b = L\,\frac{1 - 1/s}{1 - 1/s^{h_t}} \tag{37}$$

Once we have b, we can differentiate the firm into hierarchical levels by dividing b by powers of s (Eq. 35). Due to rounding errors, the sum of the employment of all hierarchical

levels may differ from the original firm size L. Any discrepancies are added (or subtracted) to the base level to give the correct firm size. The number of managers M is then simply the sum from hierarchical level h_m to h_t.

I An Agrarian Model of Institution Size

In this section, I use an adaptation of the hierarchical firm model (used in Fig. 7 of the main paper and discussed in Appendix H) to explain the institution size limits posed by an agrarian economy. In agrarian societies, the *vast* majority of the population is directly engaged in agricultural activities — a direct result of low agricultural labor productivity. This model aims to demonstrate that the large size of the agricultural population places inherent constraints on agrarian institution size. The model makes the following assumptions:

1. All agrarian institutions are 'ideal' hierarchies with the same span of control.

2. The agricultural population forms the bottom hierarchical level of *all* institutions.

3. Agrarian institution sizes are distributed according to a power law.

The model is depicted graphically in Figure 18. In formulating this model, I have in mind a feudal society in which the institutional unit can be loosely thought of as the feudal manor. These institutions are organized around the extraction of an agricultural surplus from peasants/serfs, and are defined by a rigid caste system (with serfs at the bottom). For the sake of simplicity, we assume that all peasants/serfs are engaged in agriculture.

There is evidence that feudal manors (like modern firms) were power-law distributed. For instance Hegyi et al. find an approximate power law distribution of serf ownership by nobles/aristocrats in 16th century Hungary [36]. Similarly, Kahan finds a highly skewed distribution of serf ownership in 18th century Russia [37] (although this distribution is better fit with a lognormal function).

Although the above assumptions may well be wrong (or oversimplifications), this model is intended mostly as a thought experiment. Figure 19A shows the modelled relation between the agricultural portion of the total population and mean institution size (with the span of control varying between 2 and 3). The prediction is that the agricultural population should decline rapidly as mean institution size increases.

In this model, the fraction of the population engaged in agriculture places strict limitations on institution size. Estimates vary on the size of this agricultural fraction of the population in historical agrarian societies. In Figure 19, I use Cottrell's estimate that 95% of the population in ancient Egypt was directly engaged in agricultural activity [38] (indicated by the red horizontal line in Fig. 19A). According to the model, this limits mean institution size to between 1.2 and 1.32 people (indicated by the grey region).

If we further assume that the modern relation between mean firm size and energy use per capita is applicable to agrarian institutions, we can make predictions about rates of energy consumption. We input the estimated mean institution size range into the firm size versus energy regression from Figure 1C (main paper) to predict a range of energy use per capita for this model society (Fig. 19B).

The predicted interval of roughly 10 to 30 GJ per capita is a surprisingly realistic range for a typical agrarian society. For instance Warde estimates that England used 20 GJ of energy per capita in 1560 [39]. Similarly, Malanima estimates that 1st and 2nd century Romans consumed between 9 and 17 GJ per capita [40].

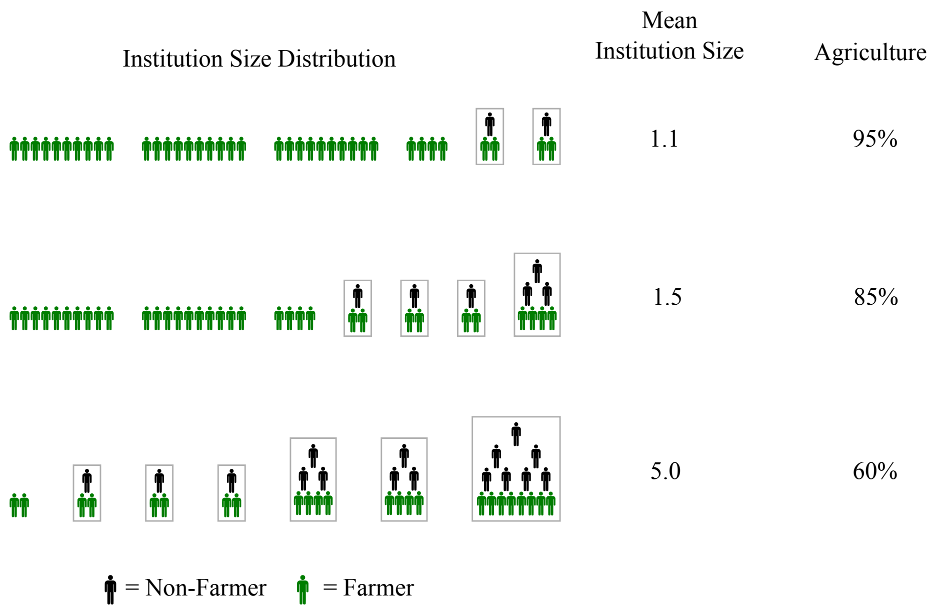

Figure 18: The Decline of Agricultural Workers as a Function of Institution Size in an Agrarian Economy

This figure shows an adaptation of the hierarchical firm model presented in Appendix H. In agrarian societies, we assume that the bottom hierarchical level of all institutions is constituted entirely of agricultural workers. As institution size increases, the relative size of the agrarian population declines. All institutions are assumed to be 'ideal' hierarchies with constant spans of control. In the model (not accurately represented here) the institution size is distributed according to a power law.

This model can be used to understand how energetic constraints place limits on institution size within agrarian societies. In all societies, the relative size of the agricultural population is a function of agricultural labor *productivity* [41]. The agriculture sector must produce a surplus of food in order to feed the non-agricultural population [42]. It follows that the fraction of workers in agriculture can decline only if their per person output of surplus food increases.

In agrarian societies, agricultural workers relied exclusively on human and animal labor, which meant that output per worker was extremely low compared to modern industrial agriculture. The result was that the agricultural surplus was very small, allowing only a small non-agricultural population to exist [43]. According to our model, this leads to inherent constraint on institution size.

Agricultural productivity, in turn, is directly related to energy use. Increasing agricultural labour productivity requires that each worker convert more energy into useful work. Historically, this meant first introducing more draft animals per worker, followed by the widespread adoption of fossil fuel powered equipment (tractors, combines, etc.). As agricultural workers increase their energy use, this will impact per capita energy use for society at large.

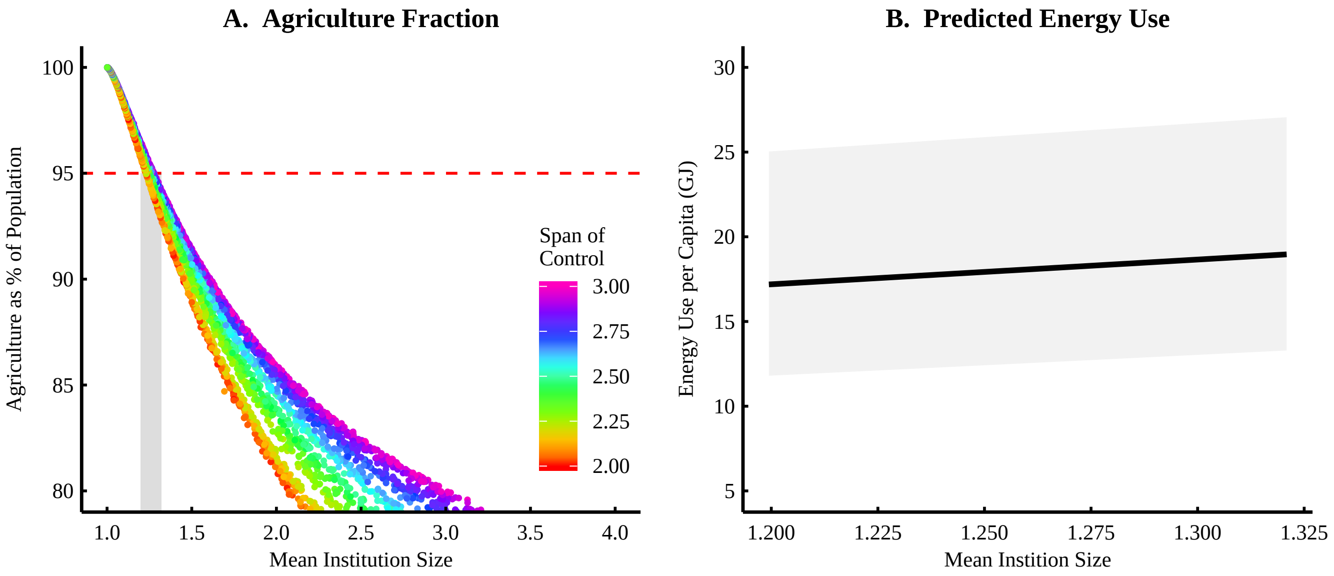

Figure 19: Modelling Agricultural Constraints on Institution Size, and the Implication for Energy Use per Capita in Agrarian Societies

This figure shows how a hierarchical model of an agrarian society can be used to relate the size of the agricultural population to institution size and energy use per capita. Panel A shows the modelled relation between the agricultural portion of the population and mean institution size. Different mean institution sizes are generated by varying the exponent of the institution size distribution. Different spans of control are indicated by color. The red horizontal line corresponds to a society with 95% of the population in agriculture, and the shaded region shows the corresponding prediction for mean institution size. Panel B shows the energy use per capita predictions for this range of institution size. These predictions are made using the national mean firm size vs. energy use per capita regression shown in Fig. 1C of the main paper. The formula is $E_{pc} = 14.3\bar{L}^{1.02}$, where E_{pc} is energy per capita and $\bar{L}$ is mean firm size. The grey region indicates the 95% confidence interval of the prediction.

Unfortunately, this model cannot be used to study the transformation from an agrarian to an industrial society because its premise *breaks down* as this transition proceeds. The model is based on a feudal society organized around the expropriation of an agricultural surplus from a serf/peasant class. As feudal relations give way to market relations, this social structure ceases to exist. New institutions form that have nothing to do with agriculture, meaning assumption 2 (the bottom level of all institutions is entirely made up of agricultural workers) becomes *absurd*.

Despite its shortcomings, this model is useful for understanding the possible limitations placed on institution size by the energetic constraints of an agrarian economy.

References

[1] Maddison A. Statistics on World Population, GDP and Per Capita GDP, 1-2008 AD; 2008. Available from: http://www.ggdc.net/maddison/Maddison.htm.

[2] Efron B, Tibshirani RJ. An introduction to the bootstrap. London: CRC press; 1994.

[3] Carter SB, Gartner SS, Haines MR, Olmstead AL, Sutch R, Wright G. Historical Statistics of the United States: Millennial Edition. Cambridge: Cambridge University Press; 2006.

[4] Nitzan J, Bichler S. Capital as Power: A Study of Order and Creorder. New York: Routledge; 2009.

[5] Ariga K, Brunello G, Ohkusa Y, Nishiyama Y. Corporate hierarchy, promotion, and firm growth: Japanese internal labor market in transition. Journal of the Japanese and International Economies. 1992;6(4):440–471.

[6] Audas R, Barmby T, Treble J. Luck, effort, and reward in an organizational hierarchy. Journal of Labor Economics. 2004;22(2):379–395.

[7] Baker G, Gibbs M, Holmstrom B. Hierarchies and compensation: A case study. European Economic Review. 1993;37(2-3):366–378.

[8] Bell B, Van Reenen J. Firm performance and wages: evidence from across the corporate hierarchy. CEP Discussion paper. 2012;1088.

[9] Dohmen TJ, Kriechel B, Pfann GA. Monkey bars and ladders: The importance of lateral and vertical job mobility in internal labor market careers. Journal of Population Economics. 2004;17(2):193–228.

[10] Eriksson T. Executive compensation and tournament theory: Empirical tests on Danish data. Journal of Labor Economics. 1999;17(2):262–280.

[11] Heyman F. Pay inequality and firm performance: evidence from matched employeremployee data. Applied Economics. 2005;37(11):1313–1327.

[12] Lima F. Internal Labor Markets: A Case Study. FEUNL Working Paper. 2000;378.

[13] Morais F, Kakabadse NK. The Corporate Gini Index (CGI) determinants and advantages: Lessons from a multinational retail company case study. International Journal of Disclosure and Governance. 2014;11(4):380–397.

[14] Mueller HM, Ouimet PP, Simintzi E. Within-Firm Pay Inequality. SSRN Working Paper. 2016;.

[15] Rajan RG, Wulf J. The flattening firm: Evidence from panel data on the changing nature of corporate hierarchies. The Review of Economics and Statistics. 2006;88(4):759–773.

[16] Treble J, Van Gameren E, Bridges S, Barmby T. The internal economics of the firm: further evidence from personnel data. Labour Economics. 2001;8(5):531–552.

[17] Hipple S. Self-employment in the United States: an update. Monthly Lab Review. 2004;127:13.

[18] Sandefur J. On the evolution of the firm size distribution in an African economy. Center for the Study of African Economies Working Paper Series. 2010;5.

[19] Hasan R, Jandoc KR. The Distribution of Firm Size in India: What Can Survey Data Tell Us? Asian Development Bank Economics Working Paper Series. 2010;(213).

[20] Angelini P, Generale A. On the evolution of firm size distributions. The American Economic Review. 2008;98(1):426–438.

[21] Cabral LM, Mata J. On the evolution of the firm size distribution: Facts and theory. American economic review. 2003;93(4):1075–1090.

[22] Gibrat R. Les inegalites economiques. Recueil Sirey; 1931.

[23] Gupta HM, Campanha JR, de Aguiar DR, Queiroz GA, Raheja CG. Gradually truncated log-normal in USA publicly traded firm size distribution. Physica A: Statistical Mechanics and its Applications. 2007;375(2):643–650.

[24] Hart PE, Prais SJ. The analysis of business concentration: a statistical approach. Journal of the Royal Statistical Society Series A (General). 1956;119(2):150–191.

[25] Stanley MH, Buldyrev SV, Havlin S, Mantegna RN, Salinger MA, Stanley HE. Zipf plots and the size distribution of firms. Economics letters. 1995;49(4):453–457.

[26] Axtell RL. Zipf distribution of US firm sizes. Science. 2001;293:1818–1820.

[27] Gaffeo E, Gallegati M, Palestrini A. On the size distribution of firms: additional evidence from the G7 countries. Physica A: Statistical Mechanics and its Applications. 2003;324(12):117–123. doi:10.1016/S0378-4371(02)01890-3.

[28] Clauset A, Shalizi CR, Newman ME. Power-law distributions in empirical data. SIAM review. 2009;51(4):661–703.

[29] Stanley MH, Amaral LA, Buldyrev SV, Havlin S, Leschhorn H, Maass P, et al. Scaling behaviour in the growth of companies. Nature. 1996;379(6568):804–806.

[30] Kaldor N. A model of economic growth. The Economic Journal. 1957;67(268):591–624.

[31] Feynman RP, Leighton RB, Sands M. The Feynman Lectures on Physics, Desktop Edition Volume I. vol. 1. Basic books; 2013.

[32] Kesten H. Random difference equations and Renewal theory for products of random matrices. Acta Mathematica. 1973;131(1):207–248. doi:10.1007/BF02392040.

[33] Biham O, Malcai O, Levy M, Solomon S. Generic emergence of power law distributions and Levy-Stable intermittent fluctuations in discrete logistic systems. Physical Review E. 1998;58(2):1352.

[34] Gabaix X. Zipf's Law for Cities: An Explanation. The Quarterly Journal of Economics. 1999;114(3):739–767. doi:10.1162/003355399556133.

[35] Harrison JM. Brownian motion and stochastic flow systems. New York: Wiley; 1985.

[36] Hegyi G, Neda Z, Santos MA. Wealth distribution and Pareto's law in the Hungarian medieval society. Physica A: Statistical Mechanics and its Applications. 2007;380(1):271–277.

[37] Kahan A, Hellie R. The plow, the hammer, and the knout: An economic history of eighteenth-century Russia. Chicago: University of Chicago Press; 1985.

[38] Cottrell F. Energy & Society (Revised): The Relation Between Energy, Social Change, and Economic Development. Bloomington: AuthorHouse; 2009.

[39] Warde P. Energy Consumption in England & Wales, 1560-2000. Consiglio nazionale delle ricerche, Istituto di studi sulle societa del Mediterraneo; 2007.

[40] Malanima P. Energy Consumption in the Roman World. In: The Ancient Mediterranean Environment between Science and History. Boston: Brill; 2013. p. 13–36.

[41] Fix B. Rethinking Economic Growth Theory from a Biophysical Perspective. Hall C, editor. New York: Springer; 2015.

[42] Giampietro M, Mayumi K, Sorman A. The Metabolic Pattern of Societies: Where Economists Fall Short. New York: Routledge; 2012.

[43] Pimentel D, Pimentel MH. Food, Energy, and Society, Third Edition. New York: CRC Press; 2007.

[44] Cleveland C, Costanza R, Hall C, Kaufmann R. Energy and the US economy: a biophysical perspective. Science. 1984;225(4665):890–897.
